# Modelling Multi-Pulse Population Dynamics from Ultrafast Spectroscopy

**DOI:** 10.1371/journal.pone.0017373

**Published:** 2011-03-21

**Authors:** Luuk J. G. W. van Wilderen, Craig N. Lincoln, Jasper J. van Thor

**Affiliations:** Division of Molecular Biosciences, Faculty of Natural Sciences, South Kensington Campus, Imperial College London, London, United Kingdom; University of Milano-Bicocca, Italy

## Abstract

Current advanced laser, optics and electronics technology allows sensitive recording of molecular dynamics, from single resonance to multi-colour and multi-pulse experiments. Extracting the occurring (bio-) physical relevant pathways via global analysis of experimental data requires a systematic investigation of connectivity schemes. Here we present a Matlab-based toolbox for this purpose. The toolbox has a graphical user interface which facilitates the application of different reaction models to the data to generate the coupled differential equations. Any time-dependent dataset can be analysed to extract time-independent correlations of the observables by using gradient or direct search methods. Specific capabilities (i.e. chirp and instrument response function) for the analysis of ultrafast pump-probe spectroscopic data are included. The inclusion of an extra pulse that interacts with a transient phase can help to disentangle complex interdependent pathways. The modelling of pathways is therefore extended by new theory (which is included in the toolbox) that describes the finite bleach (orientation) effect of single and multiple intense polarised femtosecond pulses on an ensemble of randomly oriented particles in the presence of population decay. For instance, the generally assumed flat-top multimode beam profile is adapted to a more realistic Gaussian shape, exposing the need for several corrections for accurate anisotropy measurements. In addition, the (selective) excitation (*photoselection*) and anisotropy of populations that interact with single or multiple intense polarised laser pulses is demonstrated as function of power density and beam profile. Using example values of real world experiments it is calculated to what extent this effectively orients the ensemble of particles. Finally, the implementation includes the interaction with multiple pulses in addition to depth averaging in optically dense samples. In summary, we show that mathematical modelling is essential to model and resolve the details of physical behaviour of populations in ultrafast spectroscopy such as pump-probe, pump-dump-probe and pump-repump-probe experiments.

## Introduction

The on-going development of high power, tuneable, multi-beam, and increasingly stable ultrafast laser sources as well as detector technology continues to generate exciting experimental applications. High power (>4 W) and 1–10 KHz repetition rate Ti:Sapphire regenerative amplifiers now allow the configuration of instruments that include several synchronously pumped optical parametric amplifiers (OPA's). It is not uncommon to combine four OPA's that provide independently tuneable output from the UV to mid-infrared, providing femtosecond and also synchronised picosecond sources. Here, we focus on the use of femtosecond spectroscopies of biomolecular dynamics and particularly the analytical power of detailed modelling of populations that interact with multiple pulses that have well known characteristics. The use of multiple ultrafast pulses in protein studies has proven to be a useful tool in revealing mechanistic information about transient species and their connectivities in proteins such as bacteriorhodopsin [1], proteorhodopsin [2], photoactive yellow protein [3], green fluorescent protein [4], [5], calmodulin [6], phytochrome [7], and small molecules like cyanin [8], acetylene [9] and carotenoids [10]–[12]. Real world experimental data from VIS-VIS-infrared pump-dump-probe experiments on phytochrome is used [7], and new corrections for the beam shape are proposed to ultimately extract physically relevant values such molecular directional information (via anisotropy). Two-pulse polarised infrared spectroscopy (such as VIS-infrared) is a powerful tool in the determination of (time-dependent) structural information. A good example is the accurate measurement of the angle between the diatom ligand and heme group in myoglobin [13]–[15]. Here we develop theory unexplored so far that describes the interaction of a second (polarised) visible pulse with a transient population. Multipulse spectroscopy has been used on phytochrome to directly test the validity of different reported connectivity schemes (i.e. a multi-exponential decay from the excited state [16], or a combination of sequential and parallel decays [17]). Accurate control of the time of arrival of the second laser pulse relative to the first pulse, allows for interactions of the second pulse with transient states or populations that may ordinarily not be observed in a conventional pump-probe experiment (if the transient decays faster than it is formed). In addition, by careful experimental design (of the timings and power densities), it is possible to distinguish between a species that directly decays to the ground state, and the formation of an intermediate prior to ground state formation. This example demonstrates that multi-pulse spectroscopy is a powerful method to map connectivity schemes, and the averaged photolysed fraction plays an important role in the distinction between two (contrasting) models. The interaction of intense laser light with matter is complex, and theory describing these phenomena is similarly intricate. In this work we focus on the latter, and we present theory that describes the finite bleach effect (not to be confused with a similar term that is associated with loss of emission due to a chemical reaction) of, single and multiple, intense polarised femtosecond pulses on an ensemble of randomly oriented particles. In the present context the finite bleach effect therefore refers to the effective orientation of a population by polarised laser pulse(s).

The advancement of laser technology goes hand-in-hand with detector development. Current high speed optical multichannel analyser devices make it possible to use dispersive detection to monitor photochemical reactions on a single shot basis and over hundreds of channels. The extraction of physical, chemical, or biochemical information from such large datasets requires extensive modelling which can be challenging and time consuming. To facilitate this process, we present a software tool that allows the modelling of correlations between reactions and their (transient) products by global analysis (described in section *Ι*). This type of analysis requires a model to be applied to the data that describes the connectivity between the transient species in addition to explicit chemical kinetics equations that describe each reaction. It is important to emphasise that knowledge of the system under study is not a prerequisite for a global analysis, and that careful interpretation of the results can lead to physical relevant information. A complete understanding of a complex (photo-) reaction often necessitates the use of multiple techniques and approaches, as some methods are better suited to (populate and) resolve one or more reaction products than others. For instance, a radical species can be resolved by electron paramagnetic resonance spectroscopy (EPR), while its spectroscopic signature in time-resolved visible or infrared spectroscopy measurements may be more difficult or even impossible to assign. In turn, the spectroscopic data provides detailed time resolved structural and dynamical information that is not resolved by EPR spectroscopy and is complementary as such. Currently, time-resolved (spectroscopic) data can be analysed by different software packages such as Glotaran [18], Origin (OriginLab, Northhampton, MA, USA), Pro-K (Applied Photophysics Ltd, Leatherhead, UK), Bio-Kine32 (Bio-Logic, Claix, France), using different approaches. The program that we have developed has several key differences and additions with respect to the existing packages: First, it includes advanced global analysis of time resolved spectroscopic data, in addition to the modelling of the finite bleach effect for experiments that include single or multiple visible pulses in the presence of population decay. The first requires a connectivity scheme that can be *graphically* designed. Moreover, direct search- *and* gradient based fitting methods can be easily applied to the data. A graphical user interface is included that eases the learning curve for global fitting. In addition, the left singular values from an SVD analysis can be fitted without user-required data manipulation, and it includes new methods that allow molecular modelling to simulate anisotropy and Gaussian beam profiles. The software is Matlab (Mathworks, Inc.) based and therefore cross-platform and transparent (the source code is available). Furthermore, documentation is supplied and all buttons give instant tips. The global analysis toolbox is extensively discussed in section *Ι*, and is freely downloadable at http://www3.imperial.ac.uk/people/j.vanthor/research.

In section *ΙΙ* we build on theoretical work on photoselection done by several groups [13]–[15], [19]–[21] and extend it to include a Gaussian beam profile and modelling of excited photolysis levels and anisotropy for multipulse experiments that only relatively recently has become possible due to technological progress. Importantly, we give graphical representations of ensemble averaged photolysed fractions and its corresponding anisotropy over a more realistic Gaussian beam profile of a real-world experiment. The introduction of a Gaussian beam shape as opposed to the generally assumed flat-top multimode configuration reveals the need for the inclusion of several corrections necessary for accurate information about the orientation of the vibrational transition dipole moment in relation to the visible transition dipole moment. In addition, we will show that the presented theory can be used to design an optimal experiment. We show that the extended theory for calculation of the finite bleach for multiple pulses in the presence of population decay is essential in order to extract the species associated spectral and kinetic information for multi-pulse experiments.

In essence, the described toolbox makes it possible to globally fit all data, or a noise-reduced subset of it (by applying SVD), and has extensions to include a direct search method in addition to a gradient based one, and makes it possible to graphically design a reaction model (via Matlab's Simbiology toolbox). Furthermore experimental parameters such as instrument response function and chirp can be extracted and the interaction of populations with multiple (Gaussian shaped) pulses can be modelled. The discussion commences in section *Ι* with a description of the theoretical foundations of the discussed global analysis program, and an overview of its capabilities is given by the analysis of a simulated dataset. The dual resonance interaction such as that of a visible laser pulse with an infrared beam is specifically treated in section *ΙΙ*, as careful design of this experiment can give directional molecular information. This is an advantage over visible pump-probe spectroscopy that is essentially blind for this information (for a linear absorber), unless multiple optical transitions having a specific anisotropy are detected.

## Results and Discussion

### Ι GLOBAL ANALYSIS

In the following section we specifically focus on the analysis of spectroscopic data, but it is generally applicable to the analysis of kinetic datasets (such as mass or pH as function of time). It is not uncommon that experiments produce large amounts of time-resolved data that need to be properly analysed and interpreted. Consequently, the analysis of such datasets is very time consuming. Global analysis is a powerful method that enables the extraction of time-independent correlations (such as a number of spectra that represent a –meta- stable transition states in a spectroscopic experiment) present in the data. This approach greatly enhances and facilitates the interpretation of observed dynamics by reducing the dimensionality of the problem, i.e. by describing data in time-independent spectra with their associated time constants. This procedure is widely used in chemometrics [22], [23] and spectroscopy [18], [24], [25]. It is important to stress that the results of a global analysis can potentially lead to an over-simplification and over-interpretation of the data, as the stochastic nature of some reactions (like radiative or vibrational transitions) may give rise to a range of time constants [26], [27]. A time constant distribution can be visualised by for instance lifetime density mapping, but this approach gives at best semi-quantitative information [28]. In most cases a single rate constant that represents the complete manifold is assumed [18], [29], and the presented work will therefore use the latter approach. Note that the use of singular value decomposition and global fitting assumes that the time resolved data consists of a linear combination of time-independent components. This requirement is not met for instance if vibrational cooling leads to a time-dependent change of a vibrational frequency, in which case this would need to be modelled explicitly [30]–[32]. However, most femtosecond infrared spectrometers do not have sufficient spectral resolution to resolve such dynamics. In addition, vibrational cooling processes appear to be significantly faster in large biomolecules as compared to small molecules in solution [1], [31], [33]. Therefore, in many cases femtosecond dynamics of biomolecules may also be approximated by global fitting and SVD provided that the possibility of vibrational cooling in the data is carefully assessed.

In this work we present a software toolbox to perform global analysis in the Matlab environment. It has a user-friendly graphical user interface making it straightforward to use, and functions in Windows, Linux and Mac. The code is available, making it transparent. The philosophy behind the design of the program is to make as much as possible use of the environment's built-in features. For instance, a reaction model describing the connectivity scheme can be *graphically* designed and the corresponding coupled differential equations are automatically generated. Secondly, it is not only possible to analyse data by a gradient based algorithm, but also with a direct search method that is much less sensitive to converge to a local minimum. In addition, time traces resulting from (weighted) singular decomposition (SVD), that evaluates the number of components and number and values of time constants present in the data, can be fitted as well. First we briefly describe the used methodology with emphasis on (ultrafast) spectroscopy. Next, an example dataset is analysed to demonstrate the toolbox's capabilities.

Although a number of software packages are available to employ global analysis on time-resolved data, we focus on including a presentation of a step-by-step guide to ensure good-fitting practice. Discrete steps that represent the minimum of analysis necessary to unravel the essential kinetics are therefore given in the supplementary information.

#### Fitting procedure

The fitting procedure uses two different methods, i.e. a gradient based and a direct search method, to fit (time-dependent) data 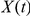 that may exhibit some degree of uncertainty. These fitting procedures are based on the calculation and minimisation of the sum of residuals using in least-squares fitting (the employed algorithm is trust-region-reflective): 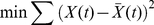
(1)


where 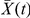 is the idealised dataset. A widely used method to minimise an objective function is the non-linear least squares analysis (see [34] for an overview on different methods). Here, a gradient based method is employed, where, the differentiability of the objective function is essential since the first derivative of the error function needs to be (iteratively) calculated. If the problem is multidimensional (when for instance multiple time-dependent experiments are available, such as at multiple wavelengths), a surface needs to be scanned for a global minimum. High local gradients can however lead to a local instead of a global minimum, which is a known problem for gradient based methods [34]. The use of different (i.e. random) starting values may be necessary for the determination of the global solution, although infinite starting values need to be scanned in theory. A direct search method can be more powerful in finding a global minimum, as it has a lower tendency of remaining in a particular (local) minimum. A grid (called a *mesh*) is built up around the current point on the objective value surface (which is *not* required to be differentiable or continuous), and consequently all directions are iteratively scanned via a pattern size that is contracting and expanding [35], [36]. If a lower objective value (i.e. the residual sum of squares as in equation 1) is encountered, the new value becomes the new current point, after which the algorithm starts again, but with an increased mesh size. The mesh size is decreased if all scanned points have a higher objective value than the current one. The employed algorithm is *patternsearch* which has proven to be very effective in global optimisation problems ranging from neural networks to peptide structure prediction [37]–[39].

#### Problem definition

In ultrafast spectroscopy exponential functions are typically used to describe first order processes such as fluorescence and transient absorption signals (note that any function, such as a power law or stretched exponential can in principle be used with the presented package):
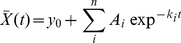
(2)


With 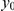 representing a baseline offset, 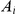 the amplitude, and 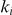 the rate constant for every 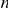 exponentials (or components) that determines the concentration (or population). In a transient absorption experiment the parameters in the formula above are therefore referred to as spectra (i.e. 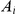), and their respective lifetimes (i.e. 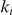). Multi-exponential decays are often observed, and it may not be obvious if these components are dependent (for instance in consecutive order) or independent (for instance depopulating in parallel) of each other. Connectivity schemes describing the physical relationship between different components or species can be very complicated, rendering the underlying coupled differential equations that describe the evolution of each species individually (given by time-dependent concentration profiles) equally complex. The approach used here is to make use of numerical solvers (the standard solver used is *ode15s*) that solve the set of given ordinary differential equations for the time-dependent concentration (or population) of each component *A_i_* in the connectivity scheme numerically (the precision that is used is 10^-6^, but can be increased to 10^−12^). The solution is produced by making use of the SimBiology toolbox. For example, the rate equation for the first component 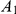 in the simple reaction 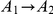 is given by the first-order rate law:
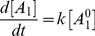
(3)


where 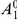 is the concentration at time zero. The concentration of all components at any given point in time equals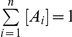, with 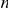 the number of components. The solution to the above differential equation for the first reactant essentially returns equation 2.

If multiple orders of magnitude in time are recorded, it is not uncommon to observe multi-exponential behaviour, making it necessary to decide on the *model* to be applied to the data. A model consists of (coupled) differential equations that are numerically solved, eliminating the otherwise necessary step of manually writing out (and implementing) the set of equations. A set of models is supplied with the program, rendering it straightforward to change example models for data analysis. Different types of models can be applied: parallel, sequential, or target (user-specified, which can include any type of connectivity scheme including equilibrium reactions). A parallel model consists of independently decaying exponential functions (representing *species*) for which the amplitudes (or *concentration)* will then be determined by global fitting. A sequential model is built from one exponential (with unit amplitude) that determines the starting amplitude of the next and so on. A target model consists of any combination of the two (including reversible reactions). Several standard models (in .*xml* format [40]) are supplied with the program, but it is possible to build and export any model by making use of the *sbiodesktop* function (part of the SimBiology toolbox) that supplies a graphical user interface which allows for the *graphical* design of a connectivity scheme. Because the toolbox makes use of the .xml format that is compatible with the Systems Biology Markup Language, it is also possible to import models from other programs that export in this standard SBML format, see [41] for more information and a list of compatible programs.

#### Extraction of time-independent correlations

The goal of the global analysis procedure is to extract the time-independent spectra and their associated rate constants, defined by the model, from the data. Importantly, the choice of model has only consequences for the resulting spectra and not for the fitted rate constants (the order of rate constants is irrelevant for the fit quality). The rate constants are shared between all measured wavelengths independently, while their associated amplitudes are allowed to vary for each wavelength. The working method of the toolbox is to generate the final spectra by first generating concentration profiles (depending on the model), and consequently fitting those to the data. The spectra (the exponential pre-factors in equation 2) and an offset parameter are calculated by QR-decomposition (via the *mldivide* function) which uses linear fitting in a least-squares sense. QR-decomposition consists of the breakdown of a matrix into an orthogonal and a triangular matrix: 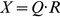
(4)


with 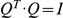 and 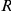 an upper triangular matrix. The solution is *unique* if all diagonal elements of *R* are positive. Because the exponential functions that are fit to the data contain linear and non-linear parameters, they are treated separately in the toolbox. The rate constants and offset in time zero are shared by all traces, and are non-linearly fitted in least squares sense with the *lsqnonlin* function.

#### Singular value decomposition (SVD)

Consider a dataset 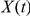 with the rows containing observables (for instance a spectrum) at each (increasing) time point. If the data for example is assumed to exhibit (multi-) exponential kinetics, a global analysis can be performed by fitting all traces simultaneously with a set of (shared) rate constants. Alternatively, a selection of time traces that are determined by SVD can be fitted (i.e. those that have the largest singular values). SVD is generally used to estimate the number of signal components (the *rank*), of which one determines a subset that can reconstruct the data via a linear combination [34], [42], [43].

The fitting of SVD traces (the left singular vectors, see below) is also used to provide the values and number of time constants in a dataset with greatest certainty, whereas the resulting basis spectra do not necessarily have physical meaning. This procedure assumes that data can be decomposed into a linear combination of unique orthogonal components by deconstructing data 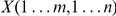 containing 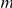 rows (corresponding to the time points) and 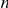 columns (corresponding to the spectra):
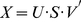
(5)


Where 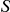 is a diagonal matrix (i.e. a matrix where all elements outside the diagonal are zero) of size 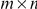 containing the singular values (in decreasing order with increasing row number), 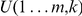 contains the traces in the time domain (the left singular vectors), and 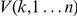 the spectral domain (the right singular vectors) that correspond to singular value 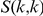. The singular values can also be used to weigh 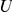 [44], [45], taking the amplitude of the singular value as measure for the dominance of the (wavelength independent) time traces: 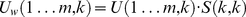
(6)


In general, the largest singular values contain the relevant signal, while the smallest represent noise. Noise can be recognised by the absence of structure in the left and right singular vectors (i.e. random in the spectral and time domain). The number of singular values to include is not always obvious, because dominant noise components might overshadow data that has physical significance. Typically, a selection is made by inspecting the fall-off of singular values of the most significant components, rejecting higher components having singular values that are in excess of a linear trend for the most significant components. The scaling of the left singular value (see equation 6) significantly increases the speed to convergence during fitting, and may further assist to identify signal over noise. The presented program allows the fitting of all data, a selection of the left singular values resulting from SVD (i.e. 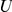), or the weighted SVD time traces (i.e. 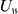).

#### Impulse response function and chirp

**Figure 1:**
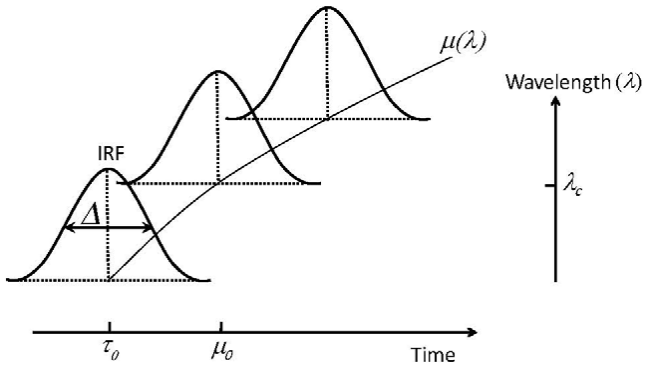
Schematic showing the IRF and chirp parameters. The IRF is represented by a Gaussian pulse arriving at 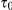 having full width half maximum 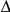, and with its intensity out of the plane of the page. Each wavelength has its own IRF, and the location (in time) 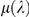 of each IRF changes as a function of wavelength (chirp). The chirp is symbolised by the curved line through the centres of each IRF, meaning that in this case the order parameter 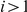 in equation 8. The central wavelength 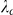 is defined as the middle of the probed spectral window, and 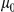 as the middle of the time domain of the IRF dominated region. Note that both 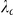 and 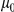 do not need to be exactly in the middle, as this depends on the order of the used IRF. The order 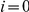 if the chirp is small (i.e. not resolved) or can be ignored.



Ultrafast spectroscopy data may exhibit a wavelength-dependence of time zero, and have a time resolution that is generally limited by the duration of the used laser pulses. The application of global analysis to these data requires therefore the modelling of chirp and impulse (or instrument) response function (IRF), respectively. The IRF, defined as the convolution of the ultrafast reaction-inducing laser pulse and the probe pulse, determines the lower limit of the shortest time scale on which a signal can be detected. If a Gaussian laser pulse (having full width half maximum 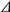 and centre location 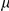, see figure 1 and equation 14) induces a response in a sample that exhibits exponential kinetics of relaxation rate 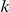, the convoluted 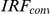 reads [18], [46]:

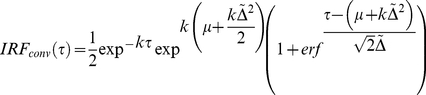
(7)With 
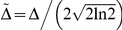
, and 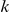 the exponential relaxation rate. The IRF is typically also distorted in time (called chirp) due to self-phase modulation and group velocity dispersion in the material in which the super-continuum (i.e. probe) is generated [47]. A long temporal chirp may prevent the detection of the fastest occurring physically relevant process in a spectral region. This is generally caused by the choice of time points (which tends to cover a logarithmic base) that effectively changes the available time resolution with wavelength. In other words, the reaction inducing pump pulse arrives at the sample at time 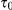, but the time of arrival of the probe pulse is delayed for every wavelength. The wavelength-dependent chirp can be modelled by a polynomial function [46]: 
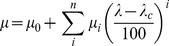
(8)


With 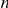 symbolising the order of the dispersion function, and 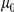 is the location of the IRF at wavelength 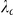. A typical experiment may exhibit a chirp in the order of tens of fs/nm, if uncompressed white light is used. The presented toolbox estimates the chirp by calculating the time shift between the signal (at each wavelength) and an instantaneous, infinitely short step function. This time delay is given by the time at which the maximum gradient occurs in the cross-correlation between each signal and the step function. The dispersion function (eq. 8) is then fitted to the resulting time delays and defines the chirp. The toolbox ultimately applies the chirp to the ideal dataset 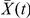 that is used to solve equation 1.

#### Example: Global and SVD analysis of model data

**Figure 2:**
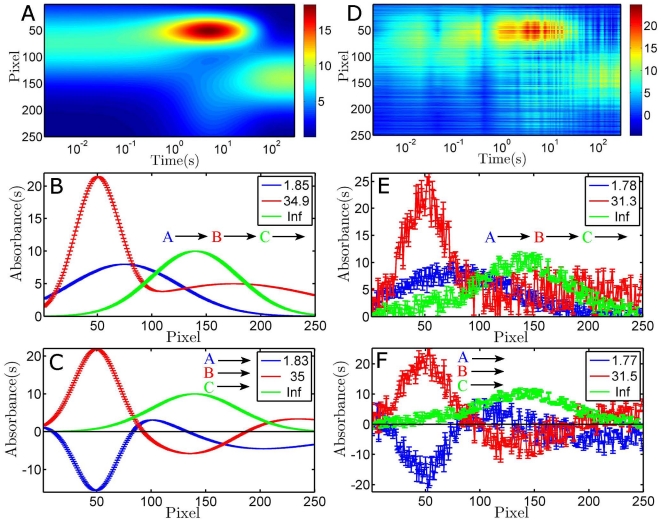
Global analysis of two simulated datasets representing transient absorption experiments. Two simulated datasets (A and D) with arbitrary wavelength (in pixels) and time delays, generated by allowing 1 species to sequentially form a second species and consecutively a final one. The spectra have (sums of) Gaussian line shapes, and the used time constants are 1.87 s and 34.8 s for the first two components to form a third (infinite or long-lived) component on a 300 second time base (91 time points at logarithmic intervals). The colour bar denotes the height of the signal. Panel D consists of the same data as panel A with ±10% added noise. Global analysis of the noiseless and noisy data is presented in panels B/C and E/F, where the pairs indicate the application of sequential and parallel models (indicated by the connectivity scheme in each panel), respectively. Typically, observation of compensating amplitudes such as those at pixel 50 in panels C and F for the 1.8 s and 35 s time constants indicates that a parallel model is unlikely to be correct. All spectra contain error bars (note that they are superimposed in panels B and C) and the legend shows the resolved time constants, including an Infinite one that is too long to be resolved on the used time base.

To show the capabilities of the program, a synthetic 3D data set is generated from three spectra (and two time constants) that sequentially evolve from one state to the next (see figure 2A). A second dataset is generated by artificially adding noise to the same dataset (resulting in the data shown in figure 2D). The noisy dataset simulates an actual experiment, and demonstrates some characteristic features of an ultrafast spectroscopy experiment which may suffer from detector noise and laser pointing instabilities that can cause spectral ‘jumps’. The data is generated by adding 10% white noise to the starting spectra (i.e. the noise is 10% of the maximum value of each ‘basis’ spectrum) that are modelled by Gaussian distribution functions, and by adding 10% white noise to the concentration profile of each component.

**Figure 3:**
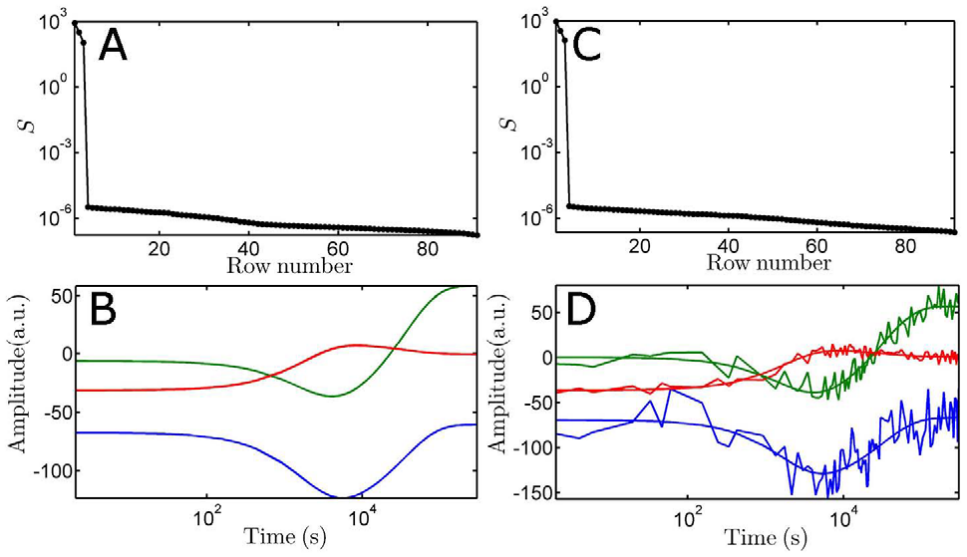
SVD analysis of the synthetic data shown in figure 2. Panels A and C show the singular values (the diagonal of *S*, note the logarithmic ordinate), which are used to get an indication of the number of occurring time constants, as function of the matrix row number (which is determined by the number of time points). Panels B and D show the first 3 left singular vectors and their corresponding fit (by using a parallel model). The fitted time constants are for panel B: 1.84 s, 34.90 s and a long-lived component, and for panel D: 1.78 s, 30.94 s and 19627 s (having errors of 0.04 s, 5.7 s and long-lived, respectively). In panel B the fits are superimposed on the data, while in panel D the fit is the smooth line through the noisy time trace.

We start the global analysis by determining of the number of components needed to satisfactorily fit the data. This is achieved by SVD analysis of the data (see above, and figure 3) that comprises the fitting of a number of SVD traces (the left singular vectors resulting from SVD). An indication of the number of relevant time constants is given by the minimum number of singular values that are needed to extract the signal out of the data, effectively filtering the noise. The noise-reducing power of SVD is illustrated by the small effect the introduced noise has on the singular values, where the noise is small. Note also that the sign of the left and right singular vectors is irrelevant for the interpretation of the data as it has no physical meaning. For both data sets 3 singular values are significantly higher than the rest (see figures 3A and C), i.e. 3 components are likely required to reconstruct the signal. Note that the fourth singular value (smaller than 10^−8^), reflects the precision of the numerical solver of the coupled differential equations that generates the linear combination of components. The white noise added to data in figure 2D is shown to be very close to being random, as the higher singular values are near those for the noise-free data. A fit of the first three left singular vectors results in time constants of about 1.8 s, 31 s and a long-lived component. A change in model from parallel to sequential has negligible influence on the quality of the fit, so only the results from the parallel model are shown in figure 3.

The time constants resulting from the fitting of the left singular vectors can be used as starting values, or fixed values if chosen, for a global analysis on the full data set, of which the results are shown in figure 2. Note that the time constants of the SVD and global analysis are very similar. Before exploring the results of the global fit in detail, some important general remarks about global fitting are discussed. Firstly, it is recommended that for global fitting the procedure should be initiated with as few parameters as possible (preferably with at least one component less than anticipated). This is particularly relevant for gradient based fitting methods (the default option in the toolbox), as convergence to a global minimum is not always guaranteed, and an unlucky choice of starting values may end up in a local minimum. The advantage of using such a conservative or restrained approach makes it less likely to ‘over-fit’ the data (i.e. by using too many components that are not present). The latter would lead to over-interpretation of the results. Critical interpretation of the results may also uncover time constants or spectra that do not necessarily have physical meaning. In practice, however, the interpretation of the results may be ambiguous, and extra care should therefore be taken to not include too many parameters. An objective measure for the goodness-of-fit is the sum of squares, which is a single number that decreases with fit quality and generally with the addition of more components. If only a small gain (i.e. a decrease) is achieved by adding an extra component, a careful evaluation of the previous fit is necessary to decide if the data is of sufficient quality to permit the inclusion of the extra component, *and* if this has any physical meaning (by investigation of their rates and spectra).

The SVD analysis shown in figure 3 has clearly indicated that not only 3 singular values are sufficient to reconstruct the majority of the signal present in the data, but also that three time constants satisfactorily fit the singular value's corresponding left singular vectors. Similarly, if three components are used to globally fit the full datasets of figure 2, it is observed that the parallel model (panels C and F) results in compensating amplitudes for the spectra that have 1.8 and 31 s time constants (look for instance at pixel 50). In general, the compensation of amplitudes of two components points to the application of an inadequate model. It could however also mean that too many time constants are used. Inspection of the sum of squares value indicates that this is not likely: it decreases from 0.093 to 0.0434 to 0.043259, for 2, 3 and 4 components (by applying the parallel model to the noisy data). Thus, a more than two-fold gain is achieved by adding one component with respect to two, while only a minor improvement is obtained by adding the fourth component (note that the number of digits chosen only illustrates the relative improvement). This strongly favours the presence of only 3 components in the analysed data. A second goodness-of-fit marker is the absence of systematic structure in the residuals in the time and spectral domain (i.e. exhibiting random noise only). Oscillations in the time domain of the residuals that do not appear random in nature indicate the need for one or more extra components. The corresponding structure in the spectral residuals gives an indication of the spectral identity of the component(s) that is (are) not optimally fit. The fits of a three-component model have random noise in the residuals (not shown), and the addition of a fourth component results in a negligible decrease in the noise of the residuals (and sum of squares). In conclusion, the global analysis gives objective criteria that favour a three-component sequential model, which is exactly how the simulated data was created. In real-life experiments additional clues may be available to judge the physical relevance of the found spectra and time constants for an applied model, and also from complementary experiments. Global analysis is an extremely useful and powerful way of dissecting, visualising and interpreting data to ultimately resolve its physical significance.

### ΙΙ ENSEMBLE-AVERAGED PHOTOLYSED FRACTION: THEORETICAL BACKGROUND

Global analysis is a powerful tool that facilitates the analysis of time-dependent data obtained from any technique. However, the next section specifically elaborates on multi-pulse ultrafast (polarised) laser spectroscopy. A general assumption in such experiments on a sample in the condensed phase is that a sample consists of randomly oriented particles. Because a laser pulse is polarised by nature it will preferentially excite those particles that have their transition dipole moment aligned with the laser's polarisation. The selective excitation has therefore effectively oriented the sample. If a second pulse arrives it will thus interact with a non-random set of particles. Here we visualise the evolution of a partially oriented population as function of time, and its interaction with a second laser pulse. We adhere to the previously used notation [7], [48] and ignore rotational diffusion between the two laser pulses. This condition is met for ultrafast laser experiments with a second laser pulse coming on a picosecond or even nanosecond timescale in some cases. Crucially, we explicitly include population decay occurring before the second pulse arrives.

The toolbox includes a graphical user interface that permits detailed insight into finite bleach effects of intense polarised femtosecond pulses, and is applicable to real world experiments. In the following discussion we focus on VIS-pump infrared-probe experiments because of the possibility to extract directional information about the vibrational transition dipole moment in relation to the visible transition dipole moment. We present a review of the photoselection theory from the literature [13]–[15], [19]–[21] and extend this theory to model experiments with multiple pulses and explicit beam profiles. By using example values of real world experiments on phytochrome [7], the calculated results are plotted to demonstrate how the excited photolysis levels of an ensemble of randomly oriented particles are determined by the power density and beam profile characteristics. We also include the shape of the laser beam, a characteristic which has previously been ignored (i.e. a flat-top multimode beam is generally assumed), and extend current theory to include anisotropy calculation for multipulse experiments.

#### Photolysed fraction

**Figure 4:**
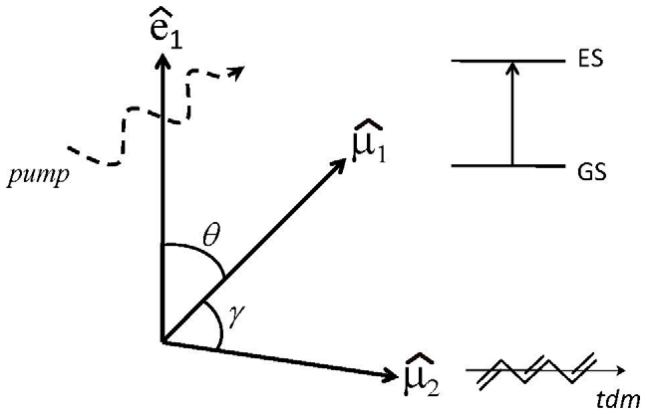
Schematic drawing defining the direction of the laser polarisation and two molecular parameters. The orientation between the pump beam polarisation 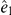 and the optical transition 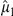 (symbolised by an energy level diagram that connects the ground and excited states) is described by 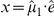, (i.e. the cosine of angle 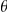). The angle 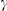 between 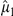 and the transition dipole moment 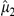 (symbolised by a linear molecule and its associated *tdm*) is fixed in space.

Before describing the time-dependent orientation distribution of a random ensemble of particles, we discuss the results for the ideal case (i.e. when the first pulse arrives). The ultimate goal is to extract structural information that gives the orientation of the vibrational transition dipole moment 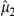 with respect to the polarisation of the visible laser 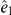. The first one has a fixed orientation relation with the optical transition along 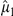 (see figure 4).

In other words, a VIS laser pulse interacts with a fraction of the randomly oriented ensemble that is governed by a certain probability distribution function, and the probe pulse, having a polarisation that is different from the pump pulse, is needed to resolve the actual orientation of the direction of the transition dipole moment [48]:
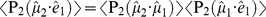
(9)


with 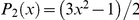 the second order Legendre polynomial, 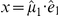 (symbolising the cosine of the angle 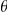 between the reaction inducing laser pulse 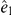 and the optical molecular transition dipole moment 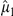), and the square brackets denoting the ensemble average over all molecular orientations. The left-hand side represents the measured anisotropy and the right-hand side the actual orientation and the optical anisotropy, respectively. The relation above explains why the measured anisotropy in pump-probe experiments where both beams have equal polarisation is always 0.4 (for infinitely low excitation power, see below), because the desired molecular information 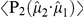 is not probed. In order to calculate the optical anisotropy, the last factor in the right-hand side of equation 9 needs to be derived first.

Consider therefore a sample with molecules that are randomly oriented. The fraction of molecules that is excited or photolysed by a laser pulse depends on the molecular properties of the particles [7], [13], [14], [48]:
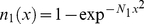
(10)


where 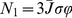
(11)


With 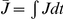 where 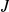 represents the total photon flux density, 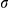 the absorption cross-section, and 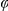 the quantum yield of the reaction. Equations 10 and 11 are only valid for a linear absorber. If the molecule is a circular absorber [13], equations 10–11 become:
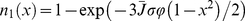
(12)


**Figure 5:**
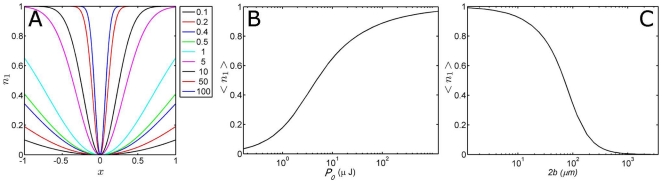
Excited photolysis fraction as function of molecular orientation, laser pump power and beam diameter. Panel A depicts the fraction photolysed molecules as function of the orientation between the optical excitation (with a flat-top, ‘multi-mode’ pulse profile) and the molecular orientation for different excitation powers (in multiples of the laser power). The ordinate represents the fraction that is photolysed for every molecular orientation. In other words, each orientation 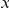 for the molecules in the ensemble relative to the orientation of the pump pulse is evaluated, and the interaction with the optical beam leads to the photolysis of a finite fraction of those molecules. Panel B shows the integrated photolysed fraction (thus for all orientations) from panel A as function of the excitation power of the laser pulse 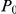, and as function of beam diameter 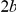 in panel C. The results shown in these panels make use of equation 15.

The power dependency of the photolysed fraction is graphically illustrated by real world experimental values of phytochrome [7], with 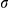 = 1.08 • 10^−16^ cm^2^/molecule, 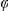 = 0.1, and a pump beam diameter of 120 µm at 640 nm with a power of 1.65 µJ (see figure 5A). These results can be generated by inserting these experimental values in the graphical user interface that is included in the presented toolbox. The resulting orientational distribution shows that a certain fraction is photolysed for every orientation 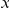, and that very high powers (in multiples of the used power) are needed to obtain full photolysis for a certain orientation. It is not possible to photolyse those molecules that have their transition dipole perpendicularly oriented (at 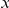 = 0) with respect to the polarisation of the laser (for a linear absorber).

The ensemble-averaged photolysed fraction 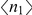 of the randomly oriented molecules can then be analytically calculated by integration or approximated by Taylor expansion:
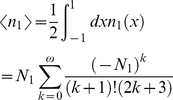
(13)


An additional illustration of the impossibility to photolyse the disadvantageously oriented molecules is shown in figure 5B (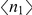 as function of 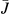) where the curve never reaches unity. If higher excited photolysis levels are needed for particular experiment where the photon flux is limiting, it is obvious that tighter focussing of the beam onto the sample increases the resulting excited photolysis levels (depicted in figure 5C).

#### Gaussian beam profile

**Figure 6:**
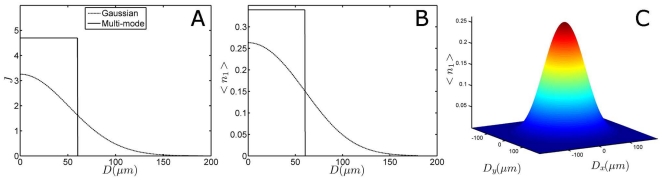
Beam profile dependence of excited photolysis fraction. Panel A shows the number of photons (x 10^20^) per m^2^ for a top-hat and a Gaussian shaped laser pulse at the used laser excitation power (1.65 µJ). Panel B depicts the resulting calculated photolysed fraction 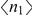, for both profiles. Both panels share the legend, and their abscissa is given by the distance from the centre of the laser beam *D*. Panel C shows how 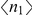
(also denoted by the colour scale) changes over a 2-dimensional Gaussian beam profile.



The calculation of the photolysed fraction generally assumes that the beam has constant power density which abruptly drops to zero at the beam diameter (see figure 6A). Laser beams are in reality more accurately modelled by a Gaussian profile *G* as opposed to a top-hat beam profile:

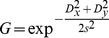
(14)In order to correct for a Gaussian beam shape, the used laser pulse power and thus 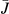 need to be multiplied by equation 14. Note that the standard deviation 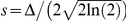, where 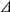
represents the full width half maximum, is the same for both the *x* and *y*-direction in the above formula, assuming a symmetric beam. Previously, a correction factor of 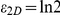
was applied to convert the maximum laser intensity of a top-hat (multi mode) profile to a Gaussian distribution [7]. This factor originates from the equality of the (two-dimensional) intensity integral of the two differently shaped pulses. The photon density over the two-dimensional beam profile (in any 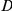-axis direction) then becomes:
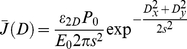
(15)


where 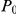 represents the laser pulse power per (beam) area and the 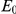 energy per photon. Equations 10, 11 and 13 for the top-hat profile can easily be modified to model Gaussian beams if 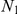 is adapted accordingly, and the results are shown in figures 6A and B for 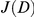 and 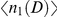 as function of the distance from centre of the Gaussian and flat-top beam profiles in one dimension. A more complete graphical presentation of the ensemble-averaged photolysed fraction over a realistic beam profile is finally shown in figure 6C.

#### Anisotropy

The analytical expressions above show that the excited photolysis level changes over the beam profile. Here we will show that, consequently, the optical anisotropy changes over the beam profile as well. In practice, an optimum anisotropy experiment consists of the measurement of three different relative orientations between pump and probe beam, i.e. parallel, perpendicular and magic angle (54.7°). The measured (macroscopic) anisotropy 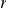 is determined by the difference in absorption in parallel 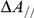 and perpendicular 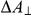 directions of the pump beam with respect to the probe beam:
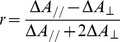
(16)


The magic angle, or anisotropy free, orientation can be reconstructed by 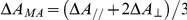. As outlined above, the anisotropy is a measure of the microscopic orientation of the polarisation orientation of the laser 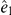, the interacting optical transition 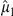, and the probed transition dipole moment 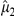 [48]. The above is valid for linear absorbers. In case degenerate transitions exist, such as for hemes [14], [15], the anisotropy is approximated by [49], [50]:
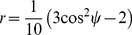
(17)


with 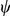 representing the angle between the linear transition and the plane in which the degenerate transition occurs (and ignoring rotational relaxation).

The ensemble-averaged factor 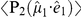, which determines the maximum obtainable anisotropy value, is approximated by 
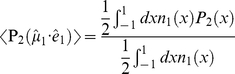
(18)


The analytical solution of this equation is a power series of which the coefficients are determined via the Cauchy multiplication rule, and for a low power density it can be approximated by the first 3 terms:
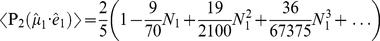
(19)


**Figure 7:**
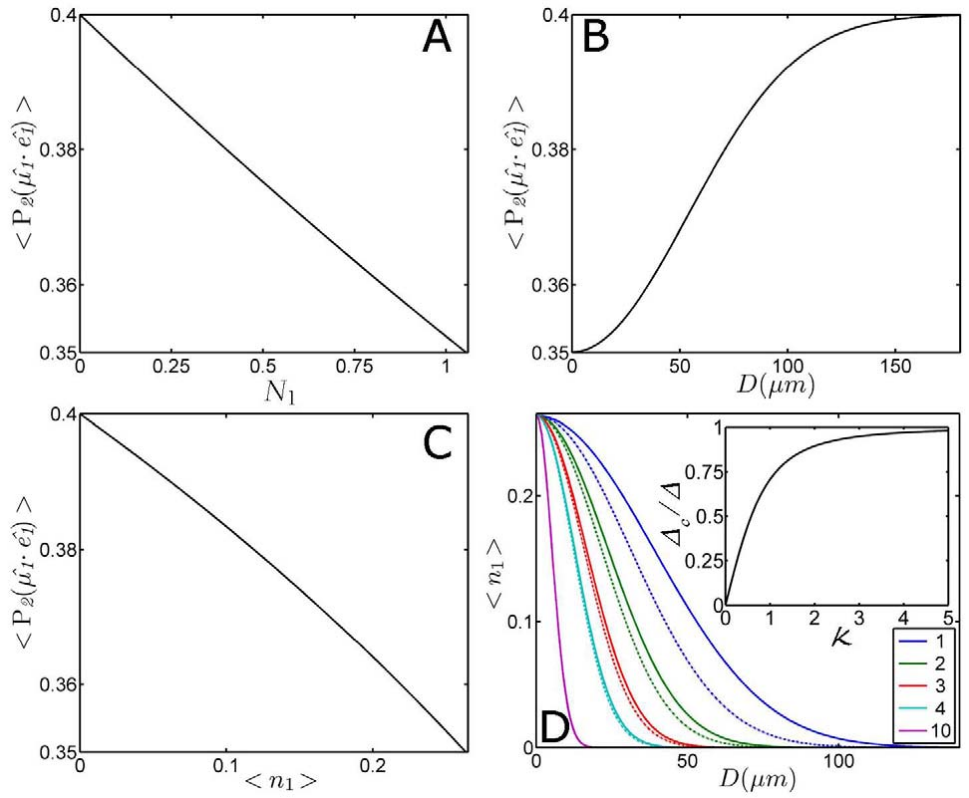
Optical anisotropy and pump/probe diameter ratio dependencies. Panels A–C show the optical anisotropy 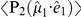 as function of power density 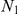, (Gaussian) beam profile, and photolysed fraction 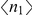, respectively. Panels A and C are beam profile independent, while panel B is only valid for a Gaussian beam, with the abscissa representing the distance from the centre of the laser beam. Panel D shows the ensemble averaged photolysed fraction as function of the beam profile with varying pump/probe beam diameter ratios (in legend). The continuous lines represent 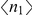, and the dashed lines 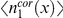. The inset shows the correction factor 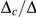
as function of the pump/probe beam ratio 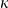.

The optical anisotropy as function of 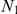 (and thus power density, see figure 7A) is thus obtained, which shows that higher powers lead to less selective excitation (i.e. if multiple relative orientations 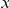
reach 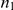 = 1, which occurs in our phytochrome experiment as described above with approximately 5 times the used laser power, see figure 5A). Ultimately these high powers lead to a reduced observed anisotropy which obviously needs to be avoided.

The substitution of equations 10, 11 and 13 and 15 in equation 19 give the relation between the optical anisotropy and the averaged photolysed fraction (see figure 7C). Note that figures 7A and C are independent of the beam profile characteristics, while figure 7B is only valid for a Gaussian profile. The flat-top profile assumes a fixed 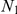 over the beam, which in turn fixes the optical anisotropy 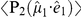. Only at very low laser pulse intensities it is possible to reach the maximum optical anisotropy of 0.4 (see equation 19). The desired molecular orientation information, defined as the angle 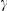 between 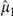 and 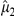, can then be calculated from the measured anisotropy (the left-hand side of equation 9), and equation 19: 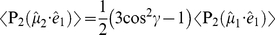
(20)


#### Beam size dependence

Earlier we have shown how extreme focussing can lead to very high excited photolysis levels (figure 5C). A pump-probe experiment consists of two pulses, each generally having a different beam diameter. Ideally, such an experiment is done with a much larger diameter of the pump beam with respect to the probe beam to obtain the largest signal. In practice, pump beams are often not more than 1.5 or 2 times larger than the probe beam, which can lead to substantial deviations from the assumed photolysed fractions (see figure 7D) and hence also from the optical anisotropy (see figure 7B). The correction factor can be calculated by the product of the Gaussian beam profile (equation 15) of the pump-and probe-beams (provided both beams overlap in time and space), which ultimately results in a correction for the photolysed fraction (in the 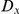-direction): 
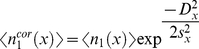
(21)


The product of the two Gaussian pump and probe beams, having ratio 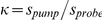, results in a new Gaussian profile (having 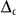) for the probe: 
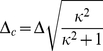
(22)


The correction factors become 0.71, 0.89 and 0.95 for 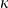 = 1, 2 and 3, respectively (see the inset in figure 7D). A reasonable value for the pump/probe beam ratio is therefore 3 or greater, in order to prevent substantial deviations of the excited photolysis level over the beam profile.

#### Depth averaging for absorbing samples

**Figure 8:**
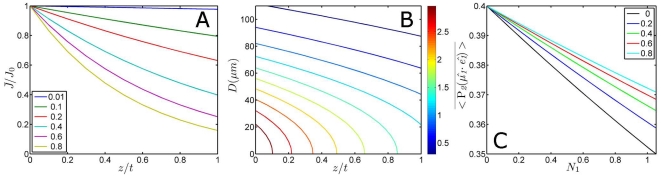
Influence of sample absorbance and thickness on photon density and depth-averaged optical anisotropy. Panel A illustrates the normalised photon density as function of the thickness ratio 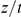 (0 represents the surface, 1 the total thickness of the sample) for different absorbances (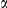 in legend). Panel B is a contour plot of the transmitted photon density 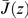 (photons/m^2^) over the thickness of the sample (having 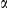) for a Gaussian beam. The colour bar denotes the height of the contours x10^20^, and the step size in height between the lines is equidistant. Panel C shows the depth-averaged optical anisotropy 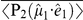 as function of 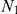 for a selection of samples with different absorbance, and for a sample without attenuation correction (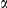 = 0).



Because the excited photolysis level changes with photon flux, it may change over sample depth as well for highly absorbing samples. A high optical density (also called optically thick) attenuates the transmitted intensity (see figure 8A), consequently changing the photolysed fraction and optical anisotropy as function of sample depth [14]:

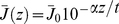
(23)With 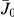 the intensity at the front of the sample, 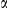 the sample absorbance, 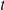 the sample thickness, and 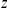 the sample depth. The attenuated intensity for a real world experiment on a sample with 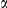 = 0.4 is shown in figure 8B.

Note that for an optically ‘thin’ sample the contours are parallel to the abscissa. The parameters 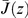 and 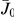 in equation 23 can be used for 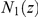 and 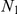 (see equation 11), allowing the calculation of the optical anisotropy via the power series in equation 19. The latter means that the excited photolysis fraction needs to be calculated for every layer, which ultimately leads to the depth-average of the optical anisotropy [14]: 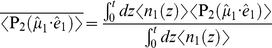
(24)


Figure 8C shows the dependency of the depth average on the laser power for different sample absorbances. As for the pump/probe beam diameter ratio and the power density, significant deviations of the observed anisotropy occur, and low powers in combination with low sample absorbances are preferred to avoid the need for corrections.

#### Multipulse spectroscopy

**Figure 9:**
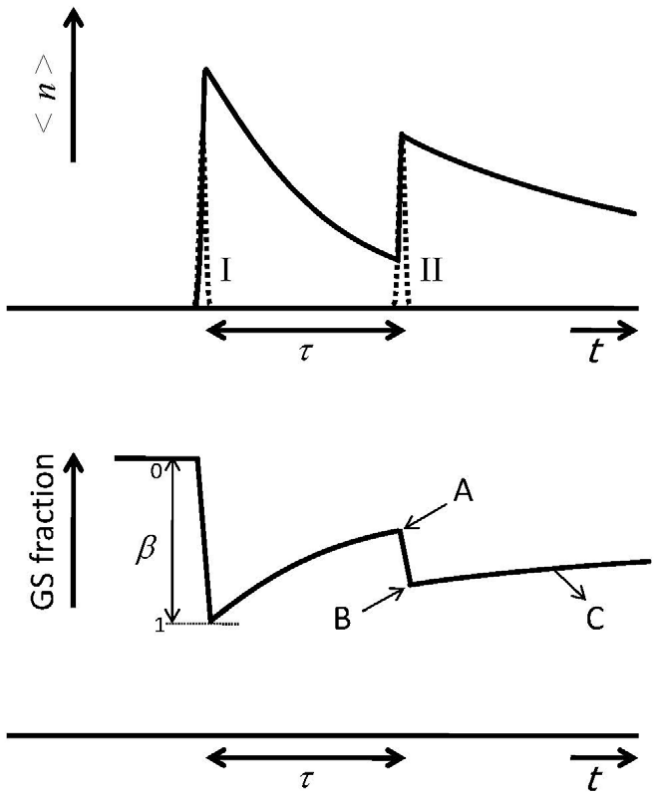
Illustration of a time course in a multipulse experiment. The top panel shows the ensemble averaged photolysed fraction as a function of time for an experiment that consists of two pulses *I* and *II* (drawn as dashed lines) that have the same power density and an inter-pulse time delay 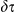. The bottom panel sketches the evolution of the corresponding ground state fraction as function of time. The recovered ground state fraction is 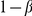 (which is normalised to the initially photolysed fraction). Detailed information about the (averaged) photolysed fraction for three cases A, B and C in a real-life experiment is shown in figure 10 (in A, B and C, respectively).

The expressions (10, 13, 18, 20, and 24) are also applicable to a multipulse experiment, such as a pump-dump-probe experiment [1], [4], [5], [7], [51], [52], of which a time course is drawn in figure 9.

If the dump pulse is also resonant with the ground state, an additional correction is needed for the photolysed fraction that has returned to the ground state at the moment the second pulse arrives. Therefore, calculation of the finite bleach requires the modelling of the population decay that occurs between pulse *I* and *II*. Here, we develop theory that specifically includes this decay. After the arrival of the first exciting pulse *I*, a certain fraction of the resonant ground state is photolysed. The second pulse *II* after delay 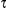
consequently excites the remaining ground state fraction. The ensemble averaged photolysed fraction for the second pulse is thus dependent on the time-dependent ground state recovery fraction 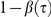 [7]:
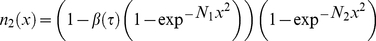
(25)


where the subscripts denote the first or second pulse. Eq. 25 is only valid if the rotational diffusion time is much longer than the time difference between the pump and dump pulses, and if the second pulse is not resonant with the higher lying excited states. From this expression it can be derived that an instantaneous second pulse (i.e. 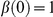) with equal excitation density gives the same ensemble averaged population as a single pulse with twice the power density (if for instance 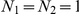 and 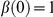 it follows that 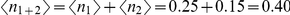, identical to 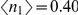 when 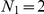). A fully recovered population (i.e. 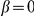) returns equation 10. The total (dump time 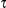 and dump power 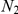 dependent) excited state population then becomes (using Taylor expansion):
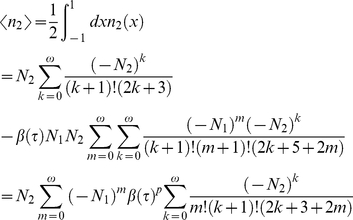
(26)


where 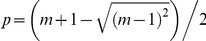. Equation 26 describes both the two-pulse as the one-pulse experiment simultaneously (most conveniently seen in the second line of the equation): with increasing dump times 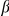 goes to zero, and 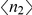 consequently forms the solution to equation 13 (i.e. 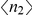 moves over the curve from 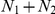 to 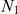).

**Figure 10:**
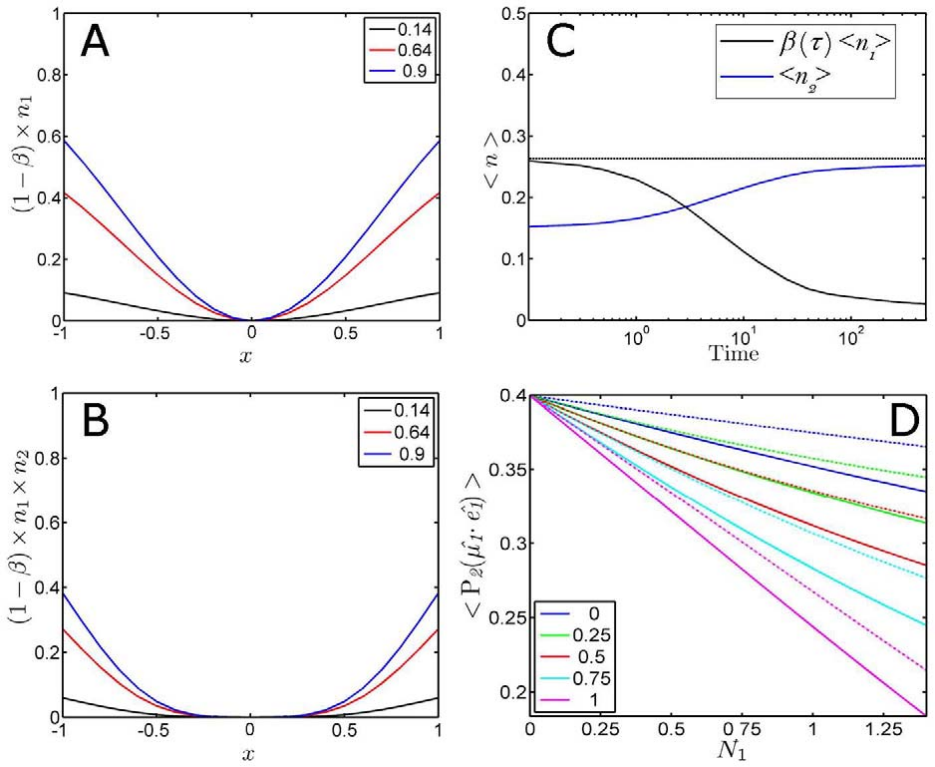
Photolysed fraction as function of orientation and time for multipulse experiments. Panel A shows 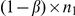 as function of 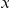, representing the recovery to the ground state of the orientation distribution of different fractions of molecules that have been photoexcited by the first pulse (case A in figure 9). Three (increasing) ground state recovered fractions (in legend) are depicted, corresponding to (increasing) inter-pulse delays of 1, 14 and 500 ps. Panel B illustrates 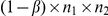 as function of 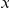, representing how the orientation distributions in panel A change after interaction with a second pulse (case B in figure 9). Panel C plots the time course of the (ensemble-averaged, i.e. 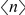) ground state recovered photolysed fraction for the first pulse, and that of the second pulse as function of the delay time 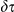 between the first and second pulse (case C in figure 9). The horizontal line is the (single-pulse) photolysed fraction at time zero. Panel D shows the optical anisotropy 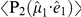 for a multipulse experiment as function of power density. Each line represents the power density dependence for a different 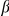 (in legend), and the continuous lines correspond to 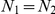 and the dashed-dot lines to 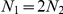.

To illustrate the use of the derived multipulse expressions a real world example is used, taken from [7]. This pump-dump-probe experiment uses the same intensity (and wavelength) for both pump and dump pulses, and three different pump-dump delay times have been used (1, 14 and 500 ps), which, depending on the *model* applied in the global analysis, correspond to different ground state recovery fractions (0.13, 0.64 and 0.9, respectively, for a parallel model). Assuming that no rotational diffusion occurs on the time scale of the delays that are used, the ground state is populated by the initially excited molecules (see figure 10A and case A in figure 9). The interaction of pulse *II* with the recovered ground stare population from case A is shown in figure 10B (corresponding to case B in figure 9). The orientation distribution of case B is very different from that in figures 5A and 10A, illustrating the necessity of the inclusion of the developed theory in similar experimental configurations. Figure 10C (black curve) shows how the averaged photolysed fraction (excited by the first pulse) evolves over time due to spontaneous decay (case C in figure 9). In our example a fraction of 0.9 eventually recovers to the ground state. Consequently, the photolysed fraction induced by the second pulse increases with time (blue curve). Note also that if both pulses arrive simultaneously, it is the excitation power of both pulses that needs to be added, and not the corresponding photolysed fractions.

The anisotropy in a multipulse experiment is analogous to equations 18 and 19, and can be calculated via:
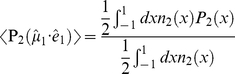
(27)


Note that the ensemble averaged excited photolysed fraction for the second pulse is dependent on the ground state recovery fraction 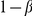. Therefore, 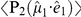 is plotted for different values of 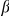 in figure 10D. Obviously, the deviation from the single pulse experiment (i.e. the continuous blue line; 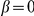) becomes extremely severe for smaller ground state recovery fractions, hampering accurate anisotropy measurements. Again, it shows the importance of the use of low power densities in (accurate) anisotropy measurements. In addition, the effect of differences in photon density is also illustrated for the case where the first pulse has twice the intensity of the second pulse (represented by the dashed-dotted lines). Note also that the curve given by 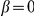 and 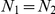 corresponds to the curve drawn in figure 7A.

For a pump-dump-probe or pump-repump-probe experiment there is usually at least some measurable cross-section for both the ground state and the intermediate at the frequency of the pulse *II*, even if it is different from that of pulse *I*. Therefore, equations 13 and 26 must be applied in order to extract the time resolved signal that corresponds to the pure intermediate 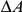, which result from a pump-probe (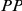), dump-probe (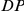) and pump-dump-probe (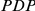) experiment: 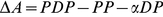
(28)


Where 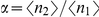. This approach has previously been applied to phytochrome [7]. The described population modelling is therefore required before the time resolved signal of a pure intermediate (just as for the pure ground state) may be analysed by global fitting and singular value decomposition.

#### Experimental implications

The theory described in this section allows for the design of an optimal laser experiment, and can extract the signal that corresponds to the dynamics of a pure intermediate that can either be an excited state (pump-dump-probe) or a ground state intermediate (pump-repump-probe). For instance, pump and probe beam diameters, optical density, and sample thickness may need to be adjusted for optimum detection of anisotropy in a polarised pump-probe experiment. Also, the photolysed fraction should be kept in the region of 0.1 if non-linear contributions are to be avoided.

#### Conclusions

Mathematical modelling and analysis is essential to resolve the details and model physical behaviour of populations in ultrafast spectroscopy experiments. A global analysis of such data can help to significantly reduce all time-dependent data to time-independent spectra and their associated time constants, and facilitating its interpretation. Here we present a software toolbox that permits the modelling of any time-dependent data by a kinetic scheme. It has been specifically developed for (dual resonance) ultrafast pump-probe spectroscopy, and includes finite bleach modelling. Based on the applied reaction model, the (bio-) physical pathways can be unravelled by globally analysing time-resolved experimental data. Throughout this work real world examples are used to illustrate molecular dynamics from pump-probe to multi-colour and multi-pulse experiments. In addition to the software, we present new theory that describes the finite bleach effect of (single and multiple) intense polarised femtosecond pulses on an ensemble of randomly oriented particles in the presence of population decay. This theory is essential in order to treat data from multi-pulse experiments, and to unravel transient populations and its characteristics in the spectral and time-domain. For single and multipulse experiments it is shown that the averaged photolysed fraction varies as function of power density and beam profile. In addition, it is graphically shown how the anisotropy changes over a Gaussian beam profile for single and multipulse experiments, and that this can lead to a significant underestimation of the actual orientation of the molecular transition dipole if compared with a top-hat ‘multi-mode’ profile. The interaction with multiple pulses in addition to depth averaging in optically dense samples is described. The modelling allows for the design of the optimum conditions for an ultrafast spectroscopy experiment. In summary, we have demonstrated that full understanding of macroscopic behaviour (aided by global analysis), requires a detailed understanding of its microscopic components (facilitated by modelling).

## Supporting Information

File S1 A real world ultrafast spectroscopic experiment on phytochrome is further discussed in File S1 to illustrate the influence of several key parameters on a multipulse experiment.
(DOC)

File S2 Documentation to the Population Dynamics Modelling Toolbox is supplied as supplementary information File S2, and includes a step-by-step guide to use the described graphical user interface, and a step-by-step guide to global fitting, describing discrete steps we consider to represent the minimum of analysis necessary to unravel the kinetics.
(DOC)
