# Supplementary material for: Modelling Multi-Pulse Population Dynamics from Ultrafast Spectroscopy
Source: PLoS One. 2011 Mar 21;6(3):e17373. doi: 10.1371/journal.pone.0017373 (PMC3061864; doi:10.1371/journal.pone.0017373)
Supplement: File S1 — A real world ultrafast spectroscopic experiment on phytochrome is further discussed in File S1 to illustrate the influence of several key parameters on a multipulse experiment. (DOC) [file pone.0017373.s001.doc]

**Supplementary information S1**

Here we illustrate the power of global analysis and the influence of selected parameters on the real world phytochrome example used in the main contribution. For instance, if the quantum yield is set to 0.7 (excitation from the ground state), and the absorption cross section[[1]](#footnote-2) is set to 3.3 x 10^-17^ cm^2^/molecule, the corresponding averaged excited photolysed fraction due to the first Gaussian-shaped pulse becomes *<n_1_>* = 0.51. The absence of exact values for cross-section and quantum yield may complicate the excited photolysed fraction calculation. In the case of phytochrome for instance, there is some degree of uncertainty about the quantum yield of ground state excitation. Several groups have determined fast sub-picosecond recombination phases [1,2], which is estimated to result in 30-40% loss of the original signal using femtosecond pulses (due to stimulated emission). However, this may be different for pulses with different spectral bandwidth, and a quantum yield of 0.7 seems therefore a reasonable estimate for the experiments described here. Therefore, we investigate to what extent the modified molecular parameters change the estimated excited photolysis levels.

With the modified parameters as outlined above, the orientation distribution function of the remaining ground state population changes, and therefore also the way a second Gaussian pulse interacts with those modified distribution. Figures 10A-C from the main contribution consequently modify to figure S1. The averaged photolysed fractions of the second pulse after 0, 1, 14 and 500 ps then become = 0.14, 0.19, 0.38 and 0.47, respectively.


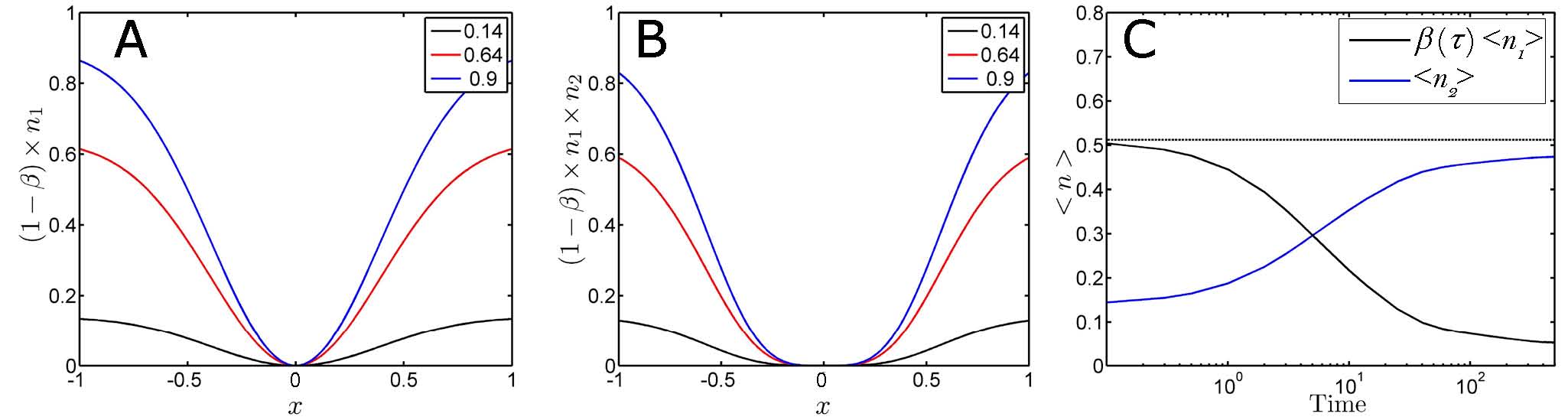
Figure S1. Photolysed fraction as function of orientation and time for multipulse experiments. Panel A shows the recovery to the ground state of the orientation distribution of different fractions of molecules that have been photoexcited by the first pulse (case A in figure 9). Three (increasing) ground state recovered fractions (in legend) are depicted, corresponding to (increasing) inter-pulse delays of 1, 14 and 500 ps. Panel B illustrates how the orientation distributions in panel A change after interaction with a second pulse (case B in figure 9). Panel C plots the time course of the ground state recovered photolysed fraction for the first pulse, and that of the second pulse as function of the delay time Δ*t* between the first and second pulse (case C in figure 9). The horizontal line is the (single-pulse) photolysed fraction at time zero.

As a result, the correction factors *α* for calculation of dump-induced multipulse experiments change to 0.27, 0.37, 0.74 and 0.93 for the corresponding delay times. The implementation of these modified correction factors to generate the dump-induced data is finally shown in figure S2, where the 1 ps dump spectra are analysed with a sum of exponentials (i.e. a parallel model, figure S2A) and a sequential model (figure S2B). The datasets for the other delay times (14 and 500 ps) show negligible differences, and are therefore not shown.


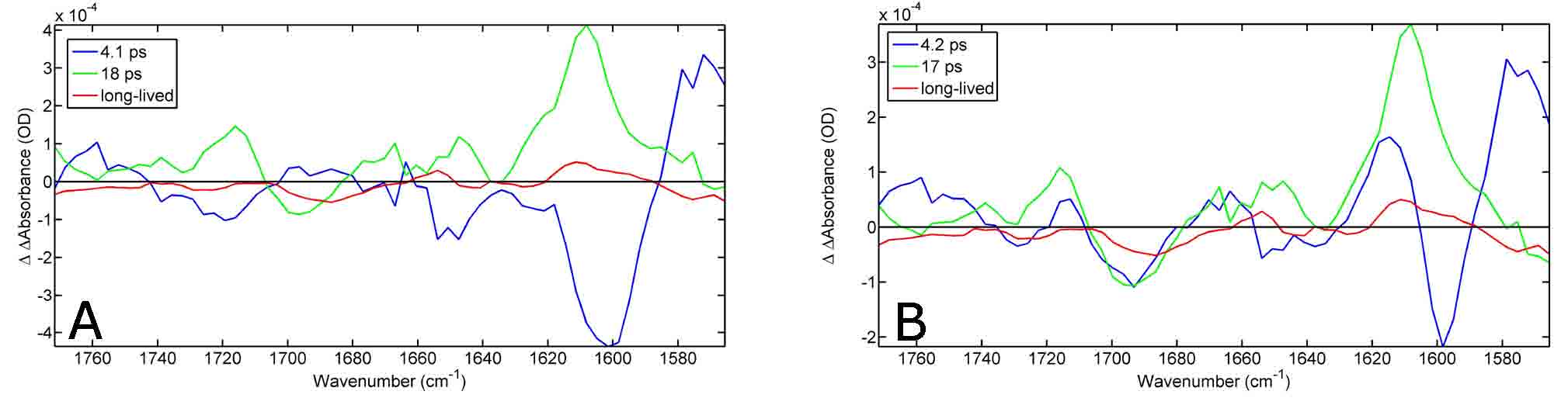


Figure S2. Global analysis results the constructed pump-dump-probe dataset with the second pulse at 1 ps. The original data is identical to data from [3] with the modified parameters discussed above. The models used are the sum of exponentials, where all components are allowed to decay with independent time constants (panel A), and a sequential model (panel B).

The first two spectra having the shortest time constants of 4 and 18 ps in figure S2A appear to be each other’s mirror image, indicating that the parallel model is not appropriate for the analysed data. The sequential model in figure S2B shows that (initially) the main absorption changes take place in the region below 1620 cm^-1^. The derivative feature in the blue spectrum that is observed in that spectral region could indicate the presence of vibrational cooling. However, the spectral shift is rather high if compared with other molecules and proteins (usually a few wavenumbers), and the associated time constant rather slow (sub-picoseconds for proteins). The structural origin of this oscillation, obtained by modifying the above molecular parameters to calculate the ensemble averaged excited photolysis levels, is unknown.

In conclusion, we have shown that detailed molecular knowledge of the studied system is important because the estimated excited photolysis levels may change significantly.

1. Dasgupta J, Frontiera RR, Taylor KC, Lagarias JC, Mathies RA (2009) Ultrafast excited-state isomerization in phytochrome revealed by femtosecond stimulated Raman spectroscopy. Proc Natl Acad Sci USA 106: 1784-1789.

2. Heyne K, Herbst J, Stehlik D, Esteban B, Lamparter T, et al. (2002) Ultrafast dynamics of phytochrome from the cyanobacterium Synechocystis, reconstituted with phycocyanobilin and phycoerythrobilin. Biophys J 82: 1004-1016.

3. van Wilderen LJGW, Clark IP, Towrie M, van Thor JJ (2009) Mid-Infrared Picosecond Pump-Dump-Probe and Pump-Repump-Probe Experiments to Resolve a Ground-State Intermediate in Cyanobacterial Phytochrome Cph1. Journal of Physical Chemistry B 113: 16354-16364.

1. The optical molecular cross-section $\sigma$ can be calculated by where $\eta$ is the molecular extinction coefficient, and $N_{a}$ the number of Avogadro. [↑](#footnote-ref-2)
