# Supplementary material for: Modelling Multi-Pulse Population Dynamics from Ultrafast Spectroscopy
Source: PLoS One. 2011 Mar 21;6(3):e17373. doi: 10.1371/journal.pone.0017373 (PMC3061864; doi:10.1371/journal.pone.0017373)
Supplement: File S2 — Documentation to the Population Dynamics Modelling Toolbox is supplied as supplementary information File S2, and includes a step-by-step guide to use the described graphical user interface, and a step-by-step guide to global fitting, describing discrete steps we consider to represent the minimum of analysis necessary to unravel the kinetics. (DOC) [file pone.0017373.s002.doc]

**­­­­­Supplementary information S2­**

**Population Dynamics Modelling Toolbox documentation**

­­­

Overview Page

1. Introduction 2
2. Setting up Matlab 2
3. Running the toolbox 2
4. Fitting demonstration 3
   1. SVD analysis 3
   2. Global fitting 12
      1. Global fitting results 15
      2. Graphical model construction 21
         1. Basic rules 21
         2. Sbiodesktop quick manual 22
5. Wavelength-dependent time shift 25
6. General Global and SVD analysis guide 33
7. *Introduction*

The Globe_Toolbox toolbox for Matlab is written in Matlab R2009b. To use the Globe_Toolbox there are no specific hardware requirements other than those required for running Matlab. The toolbox depends on several Matlab toolboxes: Simbiology, Optimization, Statistics, and the Genetic Algorithm and Direct Search Toolbox (only required if using *patternsearch*). In addition to that you need the Symbolic Math toolbox for the Population Dynamics Modelling toolbox. This documentation describes how to get started, and how to (re)produce the fits of the supplied simulated and measured ultrafast datasets (sections 4-5). These sections are written such that each section can be followed without having followed the other section in question. Next, a step-by-step guide to global fitting is described (section 6), consisting of discrete steps that we consider to represent the minimum of analysis necessary to unravel the kinetics present in a time-dependent dataset. The theoretical foundations are described in detail in the main contribution of this work.

1. *Setting up Matlab*

Copy all supplied Globe_Toolbox files to a new directory, and properly index this location and its subfolders for Matlab with File>Set Path: Add the subfolders you just copied Globe_Toolbox to and Save. Type Globe_Toolbox in the Command Window, and press enter to execute. Check if the provided Help files are correctly installed by pressing the Help button in the started GUI. If the documentation is not found, press Matlab's Start button>Desktop Tools>View Start button configuration files>Refresh start button. Check if the Globe_Toolbox is listed (referring to the correct location of the info.xml file). If the toolbox is not listed, try restarting Matlab, and check again if it is listed. Press Close. NB. There might be a problem with Windows (Vista) because of folder permissions (if next message appears: 'Matlab cannot save changes to the path' or something similar). Save 'path.def' to your startup folder, and future sessions will know where to look. You can set your startup folder in Windows by appropriately changing the 'start in' box of Matlab's shortcut properties (which you then need to use to start a new Matlab session).

1. *Running the toolbox*

To start the main graphical user interface for global fitting, type

*Globe_toolbox_v1_00*

in the command window and press execute. It may be possible that one or more characters appear clipped. This is caused by the resolution of your screen. You can adjust the window’s size by pulling its corner or sides. These settings can be permanently stored for future fitting sessions by typing:

*open Globe_toolbox_v1_00*

in the command window. Look for ‘startWindow’, and set the ‘Position’ values so that they match your screen. The numbers correspond to the pixel coordinates (in relation to the origin which is defined as left bottom of screen) with [x(start) y(start) x(width) y(width)].

To start the gui for population dynamics modelling, type

*Ave_Phot_Frac*

in the command window.

1. *Fitting demonstration*

In this section is explained how to perform a global analysis of a dataset by showing screenshots from the graphical user interface for each individual step. Two approaches are taken to analyse a dataset (see also section 7): the fitting of the left singular vectors calculated by singular value decomposition (SVD), and a simultaneous analysis of all individually collected time traces (a global analysis).

- 1. *SVD analysis*

The start of this demo is identical to the 'Start fitting demo'.

Start the toolbox (by typing inside the command window, see below), and press enter. The main graphical user interface will open. Note that figure numbering starts at 3 because this manual is part 2 of the supplementary information to the main PLOS ONE article.


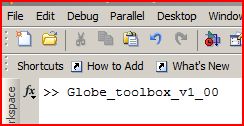


Figure S3. Screen grab of the Command Window where the GUI can be started.

Load your dataset.


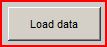


Figure S4. Screen grab of a section of the GUI.

And select the sim_data.dat dataset.


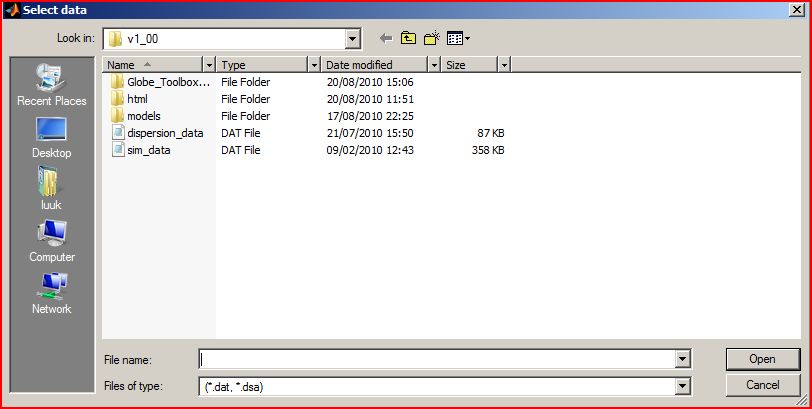


Figure S5. The dialog window that allows for data file selection.

Two figures open. The raw dataset in three dimensions (which can be rotated in 3D) and another plot that allows for data exploration. Clicking once inside the latter figure (the surface plot, see below) to activate a cross-hair, and a second time to select a point inside the surface plot. The selected point (the centre of the cross-hair) determines which time trace and which spectrum to plot individually. Click again for another point.


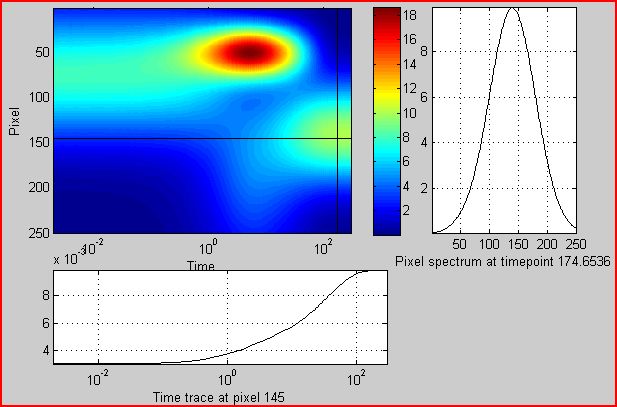
Figure S6. The raw data explorer window. Activate a cross-hair to select a slice of the raw data. The colour bar symbolises the signal size.

Define a number of rate constants in descending order (to get increasing time constants), separated by spaces to fit the left singular vectors with (the time courses, produced by SVD, that represent the dominant time components present in the data, see below). For convenience an ‘Order’ button is made to arrange the rate constants. The ‘Grab rates’ button takes the values of the last fit and puts them in the rate constants box. This only works if a fit session has already been done.


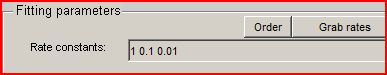


Figure S7. Screen grab of the GUI where the fitting parameters are defined. Give here the starting values for the rate constants (the inverse of the time constant) in descending order. Use the ‘Order’ button to do this automatically, and the ‘Grab rates’ to use the optimised values from the last fit.

Select the parallel model to apply to your data. It will appear in the information panel after having pressed the fit button. Note that sequential (one component forms the next and so on) and parallel (all components decay independently) model files are supplied, allowing easy fitting (just click on the model of your choice) for one up to six rate constants. The number of rate constants should thus match the number defined in the model file.


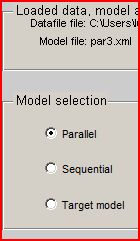


Figure S8. Screen grab of GUI where the model to be applied to the data needs to be selected.

Fix time zero to start with to prevent poor convergence: activate constraints, and check the corresponding box. Release it after having fitted your first session if desired.


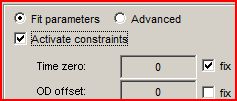


Figure S9. Screen grab of the GUI where constraints can be applied. Fix a parameter by checking the box next to the parameter.

Check the SVD box, and set the number of components to 4. This number represent the number of singular values (see below). SVD deconstructs the data into unique orthogonal vectors that have a corresponding singular value: ***SVD = U S V'*** where ***U*** are the left singular vectors (LSV) representing the time evolution, and ***V*** the right singular vector, representing a basis spectum that corresponds to singular value ***S***. The singular values are sorted in decreasing order, and the highest value is called the most 'dominant'. Use only those singular values which can reconstruct the data reliably (see below). For now, use 4 singular values.


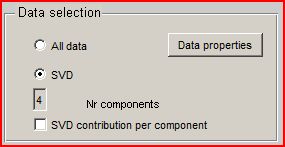


Figure S10. Screen grab of the GUI which specifies whether the full data is used for the fit, or a number of left singular vectors.

Press Fit!


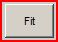


Figure S11. Screen grab of the GUI with the ‘Fit’ button.

The command window shows the fit results, with the first 4 (as selected) singular values. Note that the first three are clearly larger than the fourth one.


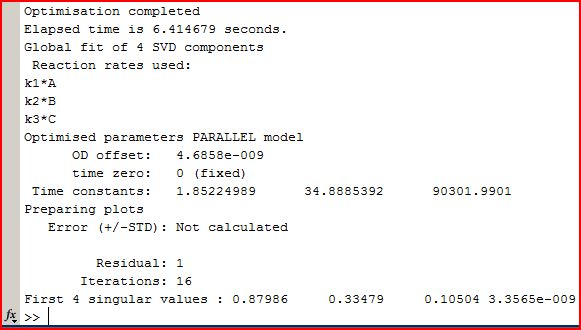


Figure S12. Screen grab of the Command Window, showing the optimised fitting parameters.

A number of figures are also generated.


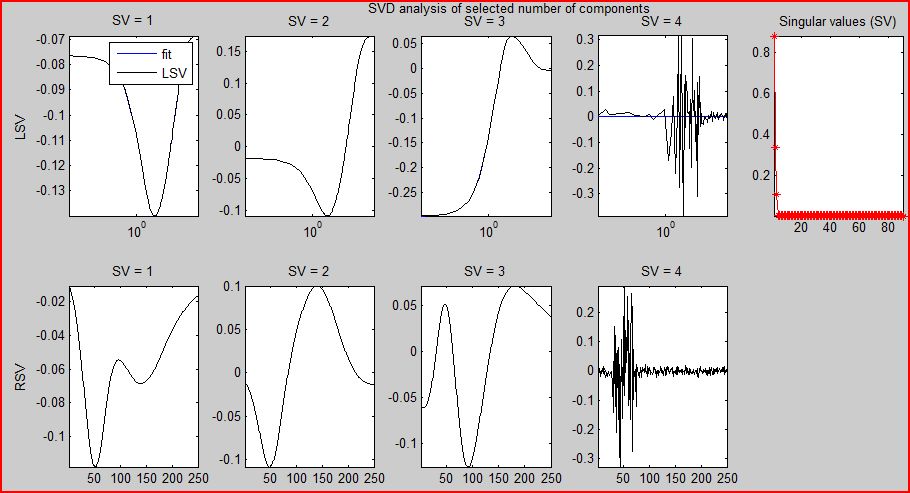


Figure S13. Screen grab of the figure generated after having performed a global fit to the left singular vectors. The top row are the LSV’s 1-4 that are fitted by the (shared) rate constants, the bottom the corresponding RSV’s. The latter are not fitted. The top-right plot show all calculated singular values.

The 'trace1' figure shows the LSV's of the first 4 (although only 4 components were selected above, the SVD is applied to the full dataset!) figures in the top row, and the RSV's in the bottom row. Besides showing the time traces (the LSV's) resulting from SVD, they are also fitted straight away (using the defined rate constants and model). It is important to know that the RSV's are not fitted or used in any way.

In this case, the fit is nearly perfect (the blue fit is on top of the black traces) with the three selected time constants. The fourth LSV basically shows a noisy trace, just as the fourth RSV (the spectrum), indicating that one singular value too many has been selected. This can also be seen in the singular value plot, where the first three singular values have an amplitude that rises from the baseline formed by components 4 to 90. That was already clear from the results shown in the command window (they are the same values). The fact that the fourth SV and its corresponding RSV and LSV are structure-less indicate that a (artificial) data set, reconstructed from SV's 1-3 and their corresponding LSV's and RSV's, is almost identical to the original dataset. In essence SVD can therefore be used as noise filter, by ignoring those SV's that are considered to represent noise.


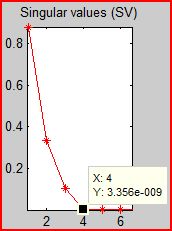


Figure S14. Zoom of the singular values shown in the previous figure. Use the magnifying glass and the ‘Data cursor’ in the figure’s toolbar for details of datapoints.

The amplitude of the exponentials used to fit the LSV's can be considered to represent a spectrum, although it has no physical meaning. Check out the ‘spectra’ figure.


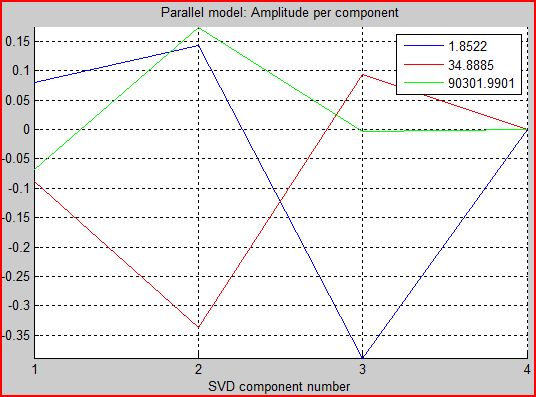


Figure S15. Screen grab of the figure (called ‘spectra’) that shows the amplitude of each exponential that has been used in the fit of each individual LSV.

This figure shows the amplitude of each exponential for each SV. For instance, it can be seen that, for the second singular value, the relative contribution of the first rate constant (the blue line) to the fit of the second LSV is higher than that of the other two rate constants (-0.336 vs. 0.1744 and 0.1435, note that the sign is irrelevant). In this way an idea is obtained of which rate constant is more strongly present in the LSV's and which less. Check also that the amplitude of the fourth component is basically zero.

The toolbox also has an additional feature, which scales the LSV with each singular value. Obviously the first SV is dominant over the second one, and the second over the third and so on. Multiplying the LSV with each corresponding SV can be done by ticking the following box.


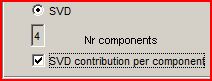


Figure S16. Screen grab of the GUI where the scaling of the LSV’s can be activated. Check the bottom box to multiply the LSV’s by their corresponding singular value.

After pressing fit again, the scaled LSV's are now fitted.


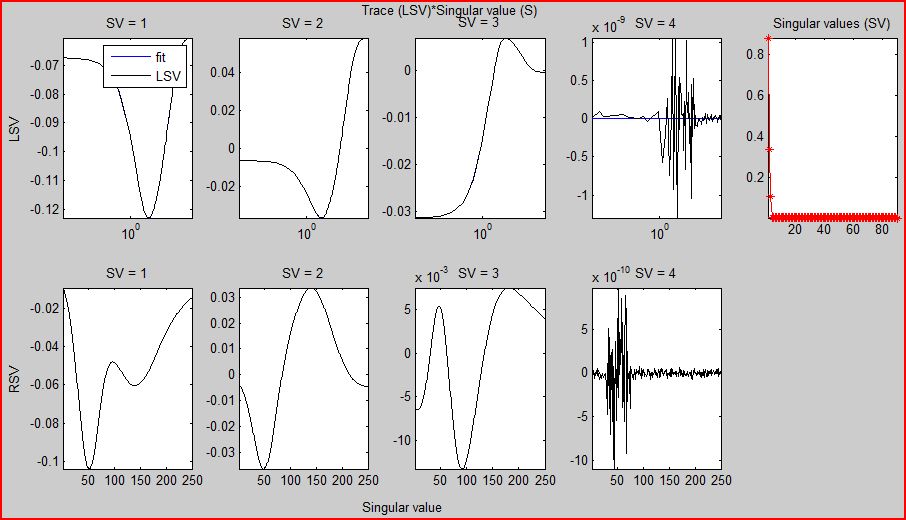


Figure S17. Screen grab of the figure generated after having performed a global fit to the left singular vectors, but with the scaling of the LSV’s activated. The LSV’s are now scaled by their corresponding singular values.

Note that the SV's and RSV's did not change, but only the LSV's. The fit results in a dramatic improvement of the sum of squares (as opposed to 1 without scaling). In general the scaling has also a significant effect on the speed of convergence (i.e. less iterations and/or less calculation time) and accuracy.


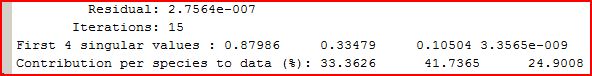


Figure S18. Screen grab of the Command Window that shows the results of a scaled LSV’s fit.

An additional plot is also shown in the spectra window. The right plot depicts the relative contribution of each rate constant to the data (still consisting of 4 SV's). This is calculated by the sum of relative contributions of each rate constant to each SV (the values shown in the left plot). Each component contributes more or less a third to the data. The percentual contribution plot can be used as an estimate of the presence of a state or species in the data, because each rate constant is linked to a specific species.


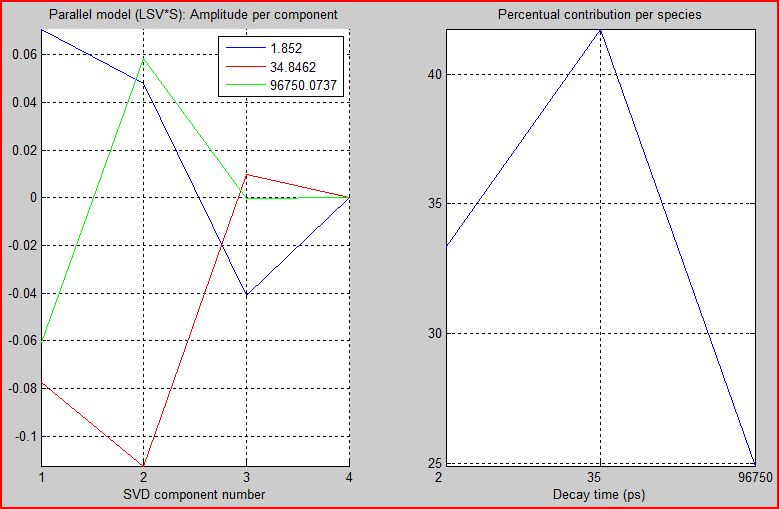


Figure S19. Screen grab of the figure (called ‘spectra’) that shows the amplitude of each exponential that has been used in the fit of each individual LSV. The right plot shows the relative contribution (determined by the amplitudes per component shown in the left plot) of each rate constant that is fitted to the data.

Finally, the fitted time constants can be used as starting values for a global fit on the full dataset.

- 1. *Global analysis*

Start the toolbox by typing *Globe_toolbox_v1_00* inside Matlab’s command window, and press enter. The main graphical user interface will open.


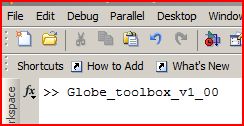


Figure S20. Screen grab of the Command Window where the GUI can be started.

Load your dataset by pressing the following button:


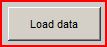


Figure S21. Screen grab of a section of the GUI.

A file dialog opens.


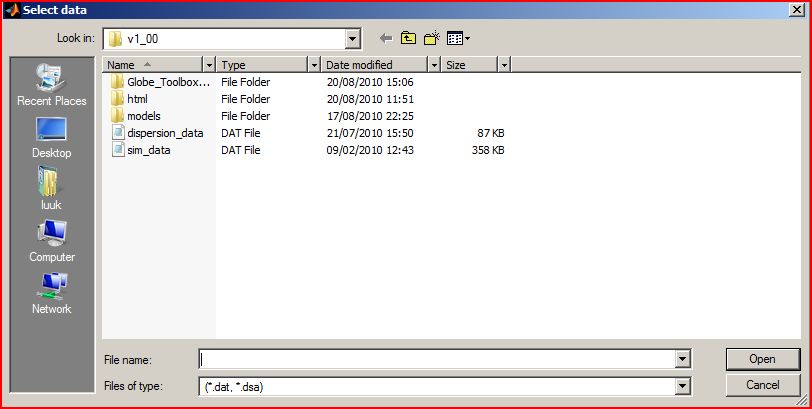


Figure S22. The dialog window that appears to select the data file.

Select *sim_data* and press enter. Two figures open, showing the raw dataset in three dimensions (it can be rotated) and another plot that allows detailed data exploration. Click once inside the latter figure (the surface plot) to activate a cross-hair, and a second time to select a point inside the surface plot. The selected point (the center of the cross-hair) determines which time trace and which spectrum to plot individually. Click again for another point.


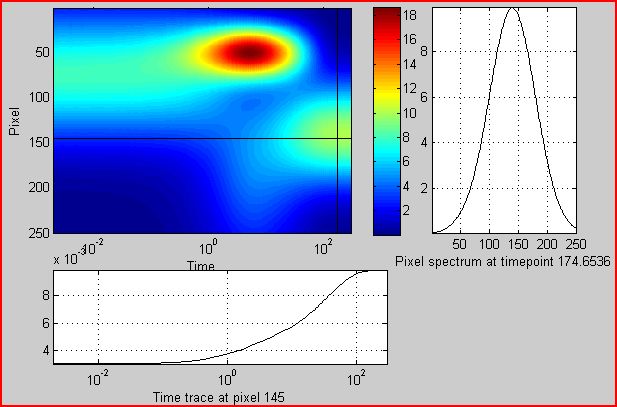


Figure S23. The raw data explorer window. Activate a cross-hair to select a slice of the raw data. The colour bar symbolises the signal size.

Next, define your starting values for the rate constants in descending order (to get increasing time constants), separated by spaces. The order button does this automatically for you. The *Grab rates* button takes the values of the last fit and puts them in the rate constants box.


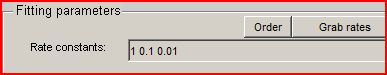


Figure S24. Screen grab of the GUI where the fitting parameters are defined. Give here the starting values for the rate constants (the inverse of the time constant) in descending order. Use the ‘Order’ button to do this automatically, and the ‘Grab rates’ to use the optimised values from the last fit.

Next, select the model to apply to your data. After selection, it will appear in the information panel after having pressed the fit button. Parallel and sequential models for up to 6 rate constants are supplied. Create and load your own connectivity scheme if desired. The number of rate constants should then match the number of transitions defined in your model.


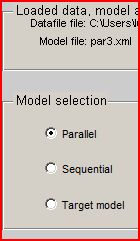


Figure S25. Screen grab of GUI where the model to be applied to the data needs to be selected.

In order to prevent poor convergence, fix time zero by: Select Activate constraints, and check the box next to the value for time zero. Release it after having fitted your first session if desired.


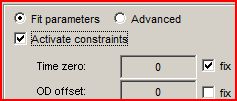


Figure S26. Screen grab of the GUI where constraints can be applied. Fix a parameter by checking the box next to the parameter.

That's it! You are ready to fit your data. Press fit!


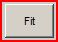


Figure S27. Screen grab of the GUI with the ‘Fit’ button.

- - 1. *Global Fitting results*

After having completed a fit, which is determined by the algorithm’s convergence criteria, a number of plots are generated. One is a three dimensional plot of the raw data.


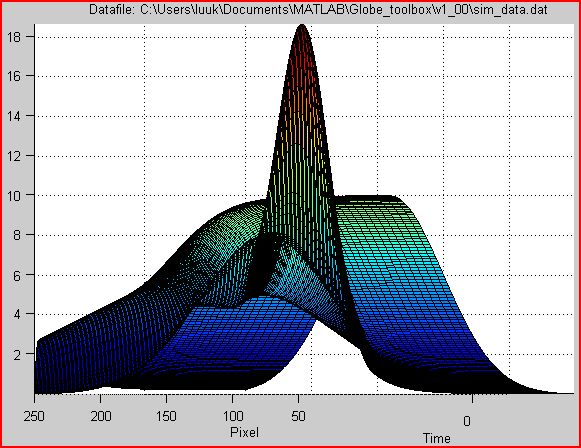


Figure S28. The raw data is plotted in three dimensions and can be rotated (the figure is called ‘Loaded_raw_data’).

Besides a number of figures, the command window gives useful information about the progress of the fit, and of the final fit results. It also indicates if convergence problems have occurred.


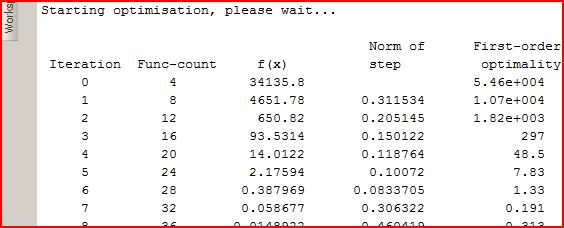


Figure S29. Screen grab of the Command Window showing the progress of the fit. The third column shows the objective value (the sum of squares) that needs to be minimised.


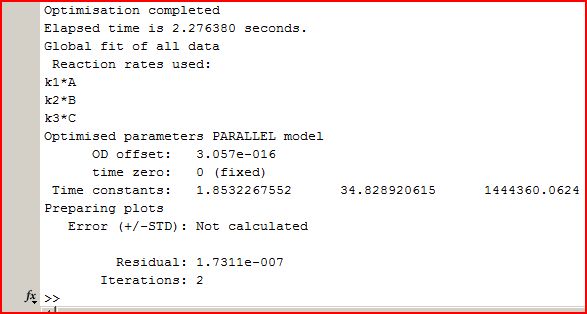


Figure S30. Screen grab of the Command Window that shows the main fitting results.

It is possible to visualise the fitting progress on-the-fly by plotting the sum of squares for each iteration. The fit can be interrupted by pressing the *Pause* and *Stop* buttons (the fit procedure terminates normally, showing all results and plots), or alternatively stopped by pressing control+C when the command window is selected (without producing any results or plots).


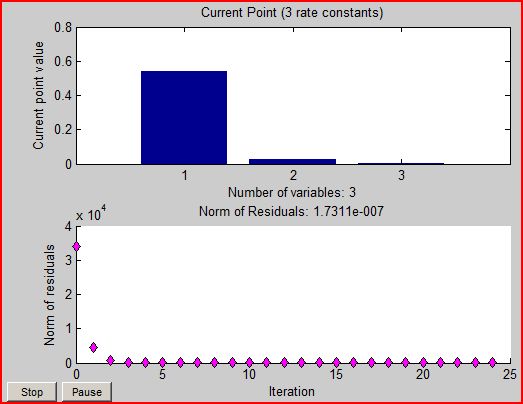


Figure S31. Screen grab of the figure (called ‘Optimization PlotFcns’) that graphically shows the progress of the fit. The top plot shows a live update of the parameters that are fitted (fixed values are not shown), the bottom plot the sum of squares. Only when this figure is shown, the fit can be interrupted by pressing the ‘Stop’ button or paused by the ‘Pause’ button. The ‘Stop’ button leads to a normal conclusion of the fit (by generating the plots), while pressing Control+C in the Command Window does not generate any plots.

Another plot depicts the fit on top of raw data (below, top panel). Patches on top of the grid (representing the raw data) are an indication that that particular region of the raw data is not perfectly fitted (a continous color plot without grid, or in other words a complete surface, represents the fit). The bottom panel shows the concentration profiles for each time constant fitted to the data. The units of the time scales are the same as those defined in your input data. The sum of squares is also given in the title of the top panel, and can be used as quality-of-fit indicator.


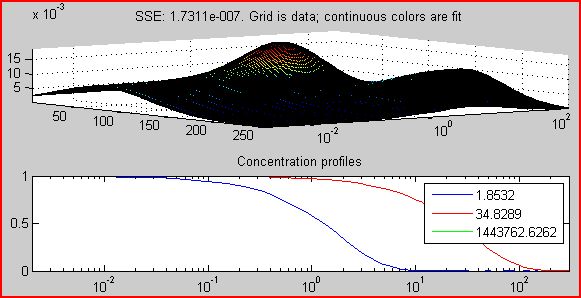


Figure S32. The generated ‘Raw data concentration profiles’ figure. The top plot shows an overlay of the raw data with the fitted data in 3D. Rotated this plot to identify regions in the data where the fit does not follow the data (appearing as patches without the grid). The bottom plot shows the concentration profiles of each component (with their corresponding time constants).

Also, up to 6x40 time traces and their fits are plotted (i.e. 6 figures that contain 40 small plots each). The raw data is drawn in black, the fit in blue. The plot title represents the index (the column index of your data file, or a channel for instance), or your calibrated value. In this case the fit follows the data nicely. Note also that the time base is logarithmic. Therefore, if the data contains negative time points, these will not be shown. Make therefore sure that your time zero is greater than or equal to zero, or shift it accordingly.


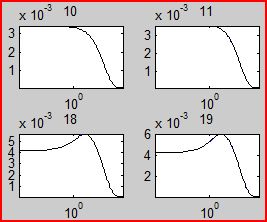


Figure S33. The individual data traces (black) are shown with their corresponding fits (blue) in the ‘trace’ figure(s). Depending on the number of traces more figures are generated, up to a maximum of 6 figures (i.e. a total of 240 traces).

It is also possible to plot all traces on a linear time base (see the plotting options panel). However, the traces with the new time base are only regenerated after having completed a new fit (not by pressing *Replot fit*).


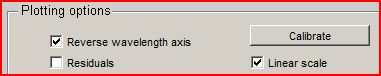


Figure S34. Screen grab of the GUI which shows a number of plot options.

The time-independent spectra are also plotted, each with its corresponding time constant (figure below, in legend). The used model is shown in the title (i.e. Parallel). It is also possible to estimate the confidence intervals for the spectra (shown in the plot) by selecting the appropriate option in the *Error estimation* panel.


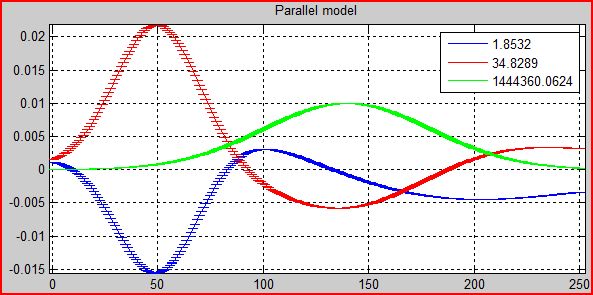


Figure S35. The generated ‘spectra’ figure showing the spectra corresponding to each time constant with the error bars.

The quality of the fit can be explored in detail by clicking once inside the generated surface plot (shown below) to activate a cross-hair, and a second time to select a point inside the surface plot. The selected point (the centre of the cross-hair) determines which individual time trace and spectrum to plot. The fit (blue line) is plotted together with the raw data (black line) for comparison.


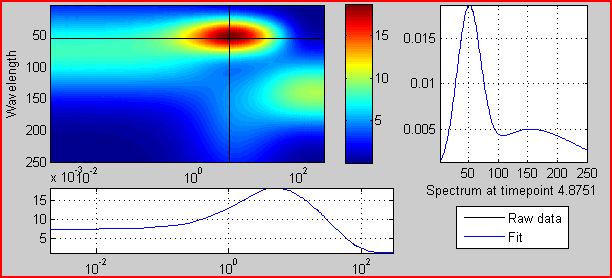


Figure S36. The generated ‘Explore fit’ figure. Activate a cross-hair to select a slice of the data that is individually plotted (the raw data in black and the fit in blue).

An overview of the quality of fit is more easily obtained by plotting the sum of the residuals in time, and the sum of residuals over all pixels. The total sum of squares is also given. Structure (i.e. non-random features such as oscillations) in the time residuals (top panel) indicate that the fit could be improved. The pixel/spectral residues (bottom panel) show then the signature of the misfit in time (top panel). In this case the dip at 1.3 seconds (the minimum in the top panel) is the dominant contributor to the spectral signature in the bottom panel. The size of the spectral residue is very small compared to the actual signal, so it seems that this fit is rather adequate, and the residuals are mainly caused by the accuracy of the solver.


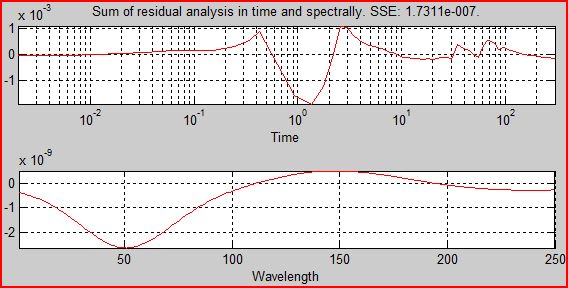


Figure S37. The ‘residuals’ figure that is generated after completion of the fit. It only appears when the ‘Residuals’ box is checked in the ‘Plotting options’ section of the GUI. The top plot shows the sum of the residuals (of all pixels) in the time domain, the bottom plot the sum of the residuals (of all time points) in the spectral domain.

- - 1. *Graphical model construction*

A global analysis makes use of a model that is applied to the data. This model describes how the different model compartments (representing for instance states, components or species) relate to each other. One component could directly form the next and so on, or two components could decay simultaneously and independently from each other. Matlab has a powerful toolbox that allows for a graphical construction of a model that can be exported (to .xml format) and consequently imported by the global analysis toolbox described here. Before we build a model, some general rules are to be considered.

- - - 1. *Basic rules*

A model consists of states or species that are connected with rate constants. These rate constants determine the speed with which the concentration or population in one state flows in to the other connected state. Some basic rules need to be obeyed when constructing a model so that the final basis spectra plotted correspond to the correct state.

- Rule 1

*Give each state a name, but the first letter should be a unique capital letter in increasing alphabetical order (i.e. A, B, etc.).* This is important because the state that has the last letter in alphabetical order has zero spectral amplitude. Obviously, this limits the number of states to 26.

- Rule 2

*The state with the last letter symbolises the finally formed state, and can be seen as a 'population sink' state.* Use this state as your last species. In the global analysis results, the amplitude of this state (representing a spectrum for instance) is not shown as it has zero amplitude. For example, consider a two-state model with rate constant *k1* (the inverse of life time) connecting states A and B. B is the last letter in the alphabet, so represents the 'sink'. A has start population 1, and flows into B which has start population 0. After some time, B has population or concentration 1, while A has 0. However, B is the last letter, so it possesses a flat line as spectrum. The results of a global analysis therefore only will show the spectrum of A. So even if A has a long-lived or infinite lifetime on the used time base, B still has zero amplitude. If the final spectrum in your data has still amplitude (which is different from the start spectrum), you can model this by introducing state C, and giving state B a very long life time.

The final state can be also be used for example to represent a 'ground state' (in difference spectroscopy this means that the original spectroscopic state has been reformed, so it will have zero amplitude since it is normalised to this state). The final state and ground state can therefore be modelled by the same state.

- Rule 3

*The rate constants have the format k1, k2,.. k10, k11, etc. up to 99* . It is important that the second (for 1-9) or second and third (for 10 to 99) characters are numbers. Typical starting values are 1, 0.10, 0.001, etc. in decreasing order.

- - - 1. *Sbiodesktop quick manual*

Launch the desktop by executing *sbiodesktop* in your command window.

Create your own model by starting a new project 'File/New Project'. Add a reaction in 'Reactions', f.i. 'A -> B' (with spaces). Use 'Mass-action' as 'Kinetic law'. Go to 'Kinetic law' tab, and make a new 'Forward rate parameter', f.i. 'k1'. Go to 'Rate species' tab, and set 'InitialAmount', for instance 1 (this is the initial concentration, i.e. a boundary value in the differential equations to be numerically solved).

Run a simulation by pressing 'Simulate/start' or Ctrl+T to check if your model works. Remember to ALWAYS do a simulation run to check if your model is working and doing what your requirements are.

You can also check out how one of the supplied models is built and use those as examples. Go to the command window, and execute for instance:

*modelobj=sbmlimport('seq2.xml')*

followed by

*simbiology(modelobj)*

The sbiodesktop opens, and you can explore the settings for the 2 component sequential model. 2 Component means that two time constants *k_1_* and *k_2_* are defined which characterise the transitions from species A → B and from B → C.

The sbiodesktop allows you to view and modify your model *graphically*. The loaded model looks like this:


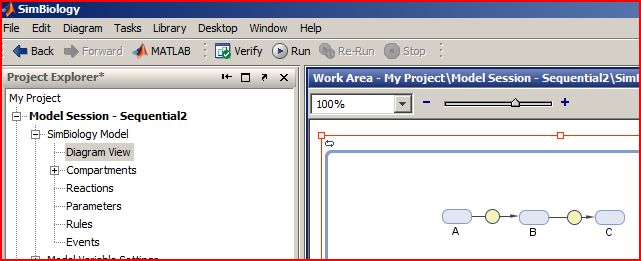


Figure S38. Screen grab of the Simbiology destop. The ‘Diagram View’ option shows the connectivity scheme of the three components A, B and C and their interdependencies. The latter is symbolised by an arrow with a yellow circle. Each item in the graphical representation can be selected and double-clicked for individual options.

Before a model is to be used in global analysis, run it first and check if the concentration profiles for each state behave as expected. The results on a 500 s (log) time base are shown below.


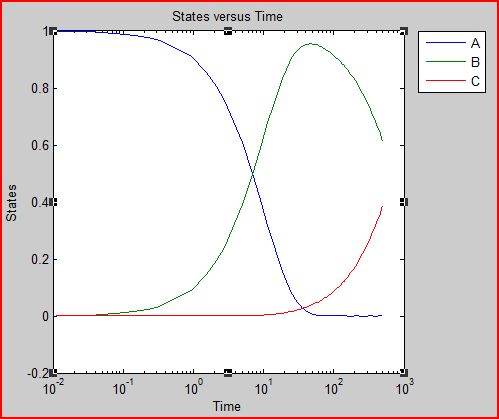


Figure S39. The concentration curves are plotted for each component. Check the ‘Model Variable Settings’ in ‘Configuration Settings’ for several calculation options.

Here the time base has been extended to 500 seconds. This and many more calculation options can be set in the ‘Configuration Settings’ window under ‘Model Variable Settings’ in your model. Export your model in .SBML format by right clicking the 'Model session' (see below) to make it useable for the global analysis package. It will actually be saved in .xml format.


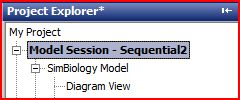


Figure S40. Screen grab of the Simbiology desktop. Right-click the ‘Model Session’ to export your model to SBML format so that it can applied to your data in a global analysis.

In a similar way more complicated models can be constructed that contain equilibriums, and combinations of parallel and sequential models. Remember to ALWAYS do a simulation run before attempting to use your model in a global analysis to ensure the model performs as expected.

1. *Wavelength-dependent time shift*

The next section describes how to fit data that exhibits a non-constant time zero for all measured observables. This typically occurs in ultrafast spectroscopy measurements, resulting in a wavelength-dependent shift of time zero. Here we show how to deal with such a dataset by analysing an actual dataset.

Load the supplied 'dispersion.dat' dataset first.


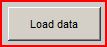


Figure S41. Screen grab of the GUI.

Two figures are generated after having selected the dataset. One is the raw dataset in three dimensions (which can be rotated) and another one that allows for data exploration. Click once inside the latter figure (the surface plot) to activate a cross-hair, and a second time to select a point inside the surface plot. The selected point (the center of the cross-hair) determines which time trace and which spectrum to plot individually. Click again for another point.

Select the advance options in the fitting parameters section, and set the starting parameters for the dispersion function.


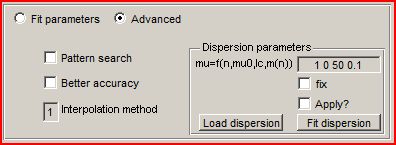


Figure S42. Screen grab of the GUI showing the ‘Advanced’ options in the ‘Fitting parameters’ panel.

The parameters are defined according to the following equation, and symbolise the order *n*, time zero μ_o_, the central wavelength λ_c_, and one extra parameter for every order μ_i_.


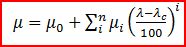


Figure S43. Phenomenological dispersion function used to describe the wavelength-dependent time zero.

Although the equation is used to describe the phenomenon, its parameters have no physical meaning. Type in some starting values (as in above screenshot), and press 'Fit dispersion'.

The command window shows the results from a robust fit to the wavelength-dependent time zero. These are calculated from the time shift that is determined by the time at which the maximum gradient in the cross-correlation occurs between an instantaneous, infinitely short step-function and each signal (at each wavelength).


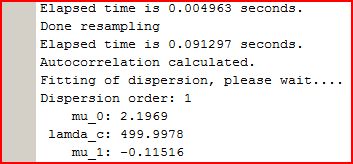


Figure S44. Screen grab of the Command Window showing the optimised results of the dispersion fit.

In addition, a figure shows the results graphically. The top panel in that figure shows the raw dataset with the superimposed dispersion curve. The same dispersion curve is shown in the bottom panel, which also includes the results from the autocorrelation determination for each pixel. It is possible to select a specific time and wavelength range for dispersion fitting, do this in the 'Data Manipulation' panel (see image below). A thick black box will appear over the raw data to show which wavelength range is selected. The specified time range determines the axis limits for the top panel.


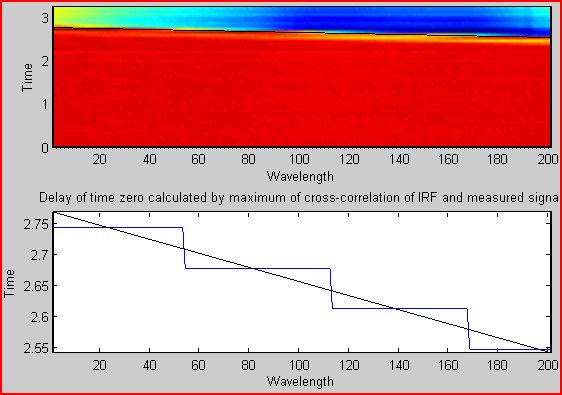


Figure S45. Figure showing the dispersion as function of wavelength. The top plot shows a surface plot of the raw data with the superimposed fit of time zero. The bottom plot shows the fit (black, the dispersion function) through the data points (blue, the time shift as described above). The steps in this plot are determined by a resampling of the time points (see below).

An important requirement of the used method is that your dataset should contain time zero. It this is not the case, the autocorrelation cannot be correctly estimated, because the defined autocorrelation function is centered at time zero. If your dataset thus starts at timepoint 500, you need to manually 'define' time zero. Two ways can be used. The easiest solution is to define it inside the dataset (shifting the time base so that it includes time zero, i.e. shifting it with -500). Obviously, this does not mean changing the time spacing. Alternatively, this can be done in the gui. Check the properties of your dataset first via the 'Data properties button'.


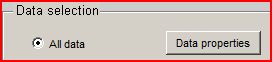


Figure S46. Screen grab of the GUI. The details of the loaded dataset can be shown in a separate window by clicking the button shown.

Any data manipulation done in the next image will be shown in the data properties box that pops up.


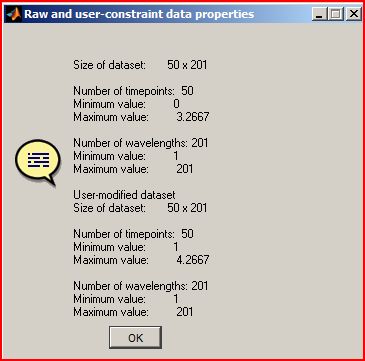


Figure S47. Figure showing the data properties of the loaded dataset. The bottom section (‘User-modified dataset’) is only shown if the data has defined constraints (see below).

The original dataset contains 0 as first time point. The modified dataset section is shown because the absolute value of 1 is added to the time base via data manipulation.


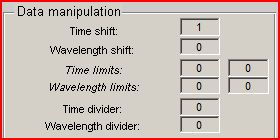


Figure S48. Screen grab of the GUI showing the ‘Data manipulation’ panel. Here basic constraints on the dataset can be applied. Check the data properties first for more details on the loaded dataset and the influence of the given constraints.

Now the dispersion cannot be estimated anymore via this method. The output now gives only a straight line.


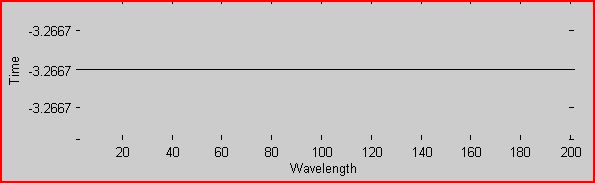


Figure S49. Screen grab of the same figure shown in Figure S45. The straight line shown is a sign that the dispersion cannot be estimated. See below for more information on how to solve this problem.

Change the 'Time shift' value back to zero. Coming back to the correct results shown above, it can be seen that the first appearance of any signal in any wavelength is at 2.55 ps. The full dispersion is 0.2 ps. Shift the whole dataset with -2.55 (ps, the time units of the dataset) in the 'Data manipulation' panel to define time zero. This is also convenient for plotting purposes later one (during global analysis), because the time traces are plotted on a logarithmic time scale by default. Otherwise a part of the signal may be absent in the plot (i.e. invisible) while it actually is included in the fit. The new fit results are now:
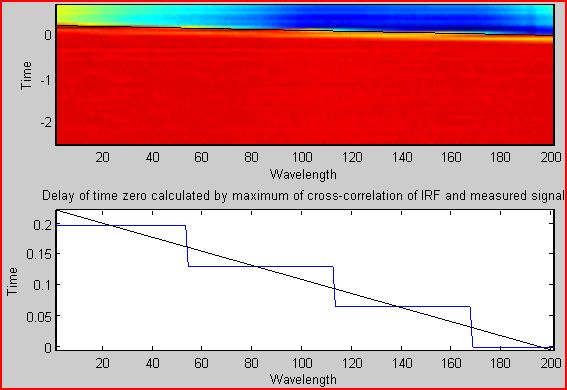


Figure S50. Screen grab of the same figure shown in Figure S45. These plots show that time zero has been successfully shifted by using the ‘time shift’ option in the ‘Data manipulation’ panel of the GUI.

The command window also shows the time it needed to perform the autocorrelation calculation, in addition to a 'resampling' time. Resampling is required since the autocorrelation can only be determined on regularly time-spaced datasets. If your dataset is regularly spaced, the resampling has no effect. The data is therefore resampled to a new time base that has the same time range as the raw data, with a time spacing that is determined by the number of time points present in the raw data. The time steps in the bottom graph of the dispersion fit results are therefor regularly spaced, and may not be optimum for your dataset. The accuracy can be improved by zooming into the time region that contains the appearance of the signal only. Count the number of irrelevant time point by using the data selector (top left symbol in image below) and the arrow keys of your keyboard to walk through the data.


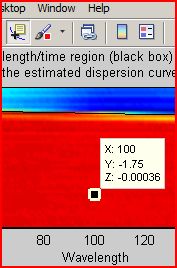


Figure S51. Zoom of the previous figure, showing that the ‘Data cursor’ (the depressed icon in the top right) can be used to get detailed info on a specific data point.

Adjust the data property settings correspondingly. Alternatively, change the data range and check the modified data properties via the 'Data properties' button. The new time limits are:


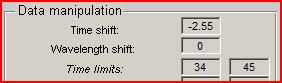


Figure S52. Screen grab of the GUI showing the modified parameters in the ‘Data manipulation’ panel.

Check the results by rotating the top graph (click in the top graph and press the rotate button). The new time range has changed the interpolation time step size, which is now slightly improved to be 0.0611 as opposed to 0.0653.


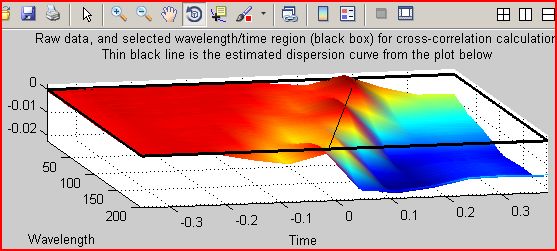


Figure S53. Detail of the fitted dispersion function. The figure is that shown in Figure S50, but rotated in 3D (press the arrow in counter-clock direction as shown above).

Note also that the dataset contains coherent artifacts (the oscillations). The wavelength-dependent time-zero seems to be qualitatively modelled. The described autocorrelation method has effectively picked the onset of the real signal from the (smaller) artifact. You can save the figure and the modelled dispersion curve via:


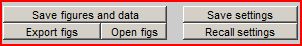


Figure S54. Screen grab of the GUI showing the top row buttons. Fitting parameters can be saved and read, and the open figures and fit results can be stored to separate files.

However, the signal attributed to artifact seems to appear earlier. If preferred, the dispersion curve properties can be manually set and fixed. After pressing 'Fit dispersion' your dispersion curve is then shown. You can also load your own dispersion curve, or the results of a previous run. The format is a single column which contains as many values as wavelengths in your dataset.


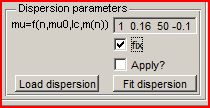


Figure S55. Screen grab of the GUI showing how to apply the fitted dispersion function to the data, and how to manually set the dispersion parameters. The loading of a previously fitted dispersion function is only possible if the fit has been previously saved by the ‘Save figures and data’ button (see Figure S54).

Finally, the dispersion curve can be used to model the wavelength-dependent time zero for your dataset in a global analysis. Press 'Apply'.


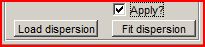


Figure S56. Screen grab of the GUI showing how to apply the fitted dispersion function to the data. Time zero will be shifted in the global analysis. The raw data is not modified.

Before starting the global analysis, modify your dataset to include the desired time and wavelength ranges, but keep the value defined in time shift. Fix also time zero to 0 (in this case it is -2.55 ps).


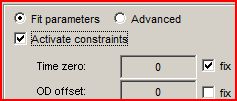


Figure S57. Screen grab of the GUI showing the ‘Fitting parameters’ section. Fix time zero by selecting the adjacent box to make sure the appropriate time zero is used.

That concludes the fitting of a pixel-dependent shift in time.

1. *General Global and SVD analysis guide*

Although many software packages are available to employ global analysis on time-resolved data, we feel the necessity to present a step-by-step guide for good-fitting practice. We therefore recommend the following discrete steps that represent the minimum of analysis necessary to unravel the kinetics. The usual format of the data is arranged in a matrix with rows containing the time points, and columns the time-dependent observables (wavelength in this case). Outliers in the data may significantly decrease the accuracy of the overall fitting and have to be excluded before proceeding to the data analysis. For most effective use of the presented toolbox, define time zero in your dataset at the time point when any signal appears first on any channel.

A global analysis can be performed by fitting all time traces at a range of different wavelengths simultaneously with a set of (shared) rate constants. Alternatively, a selection of the data can be fitted, i.e. a minimum number of left singular vectors (determined by singular value decomposition, SVD, see main text) that describes the data sufficiently. The latter method is generally used to estimate the number of signal components (the *rank*), and effectively functions as a noise filter. We start with an SVD of the data, and use this to determine the number and estimate the value of rate constants present in the data (start for instance with 4 components; step 1A). A global analysis of the whole dataset gives very similar results, but this is described in step 2. Compare both methods, or start directly with step 2 if preferred. By plotting the singular values calculated by SVD, we determine how many of these values significantly lie above the bulk (see figures 2A and C for an example where 3 values are clearly dominant), and consider only the largest values for further analysis. Plot also the corresponding left singular vectors (the columns in *U*), and the right singular vectors (the rows in *V*) that represent the corresponding time and spectral profiles, respectively. If desired, fit the time traces *U*, as they generally give a good estimate of the time constants present in the data (step 1B). The described fitting procedure of time constants to the left singular vectors is similar to global analysis on the full dataset (see below). A faster convergence to a minimum is generally achieved by using weighted SVD (*U_w_*), where each left singular value is multiplied by its corresponding singular value. SVD mathematically decomposes the data into orthogonal components, and the resulting ‘spectra’ *V* do not have physical meaning. Check if the time trace of the smallest of the dominant singular values can be attributed to signal (i.e. having non-random structure), and if the next singular value can be discarded (i.e. showing random noise). Simultaneously, do this for the spectral profile as well (step 1C). Ignore those components (corresponding to the smallest singular values) that appear to be random. This procedure is not always clear-cut, as a small signal may still be hidden in larger singular values that represent noise. Only a global analysis on the full dataset, where the resulting spectra may have physical meaning, can be used to extract all signal components. In this case, compare the fitting quality (determined by the sum of squares –lower is better- and the residuals in time and wavelength –smaller and without structure is better-) for the time constants and corresponding spectra with and without an extra component in the global analysis, and determine if the resulting extra spectrum has physical meaning. After having decided on the number of signal components in the data, perform the global analysis on the full data set (step 2A). If an estimate of the occurring time constants is obtained, use these values as starting values for the global analysis. Before you start fitting, visually determine time zero and fix it. If time zero is wavelength dependent dispersion correction is needed first (see step 2E).

To gain insight into the rich kinetic information from the time-resolved data, we begin by employing a simple parallel model, where each component is allowed to decay independently, and each (difference) spectrum at progressing time consists of the sum of these time-independent spectra. Make sure you start with at least one component less than determined by the SVD procedure in step 1, to make the fitting process faster, prevent over-fitting, and yet obtain a good idea about the spectral evolution. Remember that fitting is an iterative process, and that usually several steps are needed for a satisfactory fit. The IRF is ignored for a moment, but if required, continue with step 2F. Increase the number of components cautiously, by using the previously optimised values (if you have followed step 1B, and organise the rate constants in decreasing order). If this is the first fitting session, estimate the rate constants from the raw data, or start with one or more rate constants that decrease in size on a logarithmic time scale (i.e. 1, 0.1, 0.01, etc.). With every rate constant addition, check if the sum of squares decreases significantly, and *simultaneously* monitor the fit to the individual traces and the structure of the sum of residuals in time and spectrum (step 2C). If the residuals appear random, or only a minor decrease in the sum of squares is observed, stop fitting and decide if the last component is needed for a satisfactory fit. A final fine-tuning step is possibly the release of time zero, if a part of the signal before the current time zero appears to be poorly modelled. If the fit fails to converge with the release of time zero, fix it again, and manually scan time for the time zero. If time zero is very different (and/or fast components are visible), go back to step 2A.

In practice the gradient-based method that is used to minimise the fit to the data works well, although this is not always the case. If for instance two of the fitted components have a tendency to converge to the same value, you can try to fix one of the two components involved, but you need to make sure that this has physical meaning. These problematic cases require a different approach. Therefore, the presented toolbox also includes the ‘advanced’ (single-click) option to use a direct search method over a gradient based algorithm (step 2D). Although significantly more calculation time is required than gradient based fitting (it scans all directions as opposed to one, see Fitting procedure in the main text), it is likely to converge to the global minimum within one fitting session. Ideally, infinite starting values need to be scanned for a gradient based method to ensure having reached the global minimum, but this is unrealistic in practice. The slow-but-accurate convergence characteristic of the direct search method forms therefore a viable alternative. If chosen, step 2A to 2C need to be followed for this method as well. Having decided the optimum number of rate constants, the next important step is to decide on the model (step 3). However, make a note of all parameters first, or, if the presented toolbox is used, save an input file (with a relevant name) that contains all relevant parameters for a future session (step 3A). Compare the results of the application of the sequential and parallel models to your data (by using the optimised values, step 3B). An indication for inappropriateness of the model for your data is the presence of compensating amplitudes, which typically occurs in sets of two (although more than two are not uncommon). If all spectra are unique and physically relevant, the fit is considered complete and its results are ready for interpretation (step 4). Compensating amplitudes for spectra (or species) may also indicate over-fitting (i.e. one component too many is used) or the occurrence of a more complex connectivity scheme that contains both parallel and sequential transitions. Reduce the amount of components and check how much the sum of square increases (i.e. a worse fit). If only a small gain is achieved by the extra component consider ignoring that component. If the additional component significantly reduces the sum of squares, try using a target model, for instance 2 components that decay in parallel while one of those sequentially forms a third component). A model can be graphically constructed with Matlab’s *sbiodesktop* (step 3B). It is possible that multiple models fit your data equally well, and that other information about your studied system is required to decide on the appropriateness of one of your models. This can be from literature, or from other performed experiments (under different conditions for instance, where one or more components/species are preferentially populated). Choose your model, or present all tried models for further interpretation (step 4).

As mentioned before, data from ultrafast spectroscopy experiments may exhibit a wavelength dependent change of time zero, and non-exponential phenomena around time zero that are time- and wavelength-dependent. Correction for changes in time zero is from now on referred to as dispersion correction (step 2E). If the dispersion is significant (can be in the order of tens of fs/nm or more), determine the extent of it by calculating the cross-correlation between the IRF (for instance a step function) and every wavelength. The dataset can then be effectively straightened out by projecting the data on a new time base such that all wavelength traces share the same time zero. Kinetics occurring around time zero may be modelled by an IRF and coherent artefacts. Both phenomena can also be enveloped by a single IRF (step 2F). Different approaches can be taken in this procedure. The most straightforward method is to use a single IRF that covers the wavelength-dependent chirp (if present) and artefacts as well. This may result in some loss in time resolution, since the highest possible time-resolution for a dataset is only achieved by modelling of the chirp. A second approach is to measure the IRF indirectly by adding a reference material (such as a dye) with known spectral properties. Similarly, contributions of the coherent artefact and IRF to the signal can be measured by a repeat experiment on water only in the exact same sample cell (without moving or modifying it in any way) and configuration as the data was collected on. Ultimately, the resulting (possibly scaled) data can then be subtracted from the sample data.
